# Deficiency of Werner RecQ-type DNA helicase causes premature malnutrition in zebrafish

**DOI:** 10.1016/j.isci.2026.114760

**Published:** 2026-01-21

**Authors:** Kota Ujibe, Makoto Kashima, Miku Kataoka, Rintaro Shimada, Masashige Okamoto, Isao Kobayashi, Seiji Wada, Hiroki Matsuda, Akira Sakamoto, Hiromi Hirata

**Affiliations:** 1 Laboratory of Brain Science, Department of Chemistry and Biological Science, College of Science and Engineering, Aoyama Gakuin University, Sagamihara, Kanagawa, Japan; 2 Department of Biomolecular Science, Faculty of Science, Toho University, Funabashi, Chiba, Japan; 3 Laboratory of Molecular Spectroscopy, Department of Chemistry and Biological Science, College of Science and Engineering, Aoyama Gakuin University, Sagamihara, Kanagawa, Japan; 4 Faculty of Biological Science and Technology, Institute of Science and Engineering, Kanazawa University, Kanazawa, Ishikawa, Japan; 5 Department of Biomedical Science, College of Life Sciences, Ritsumeikan University, Kusatsu, Shiga, Japan

**Keywords:** Developmental biology, Pathophysiology

## Abstract

Werner syndrome is a genetic progeria characterized by premature aging symptoms, but its early-onset pathology remains unclear. We generated *wrn* truncation mutant (*wrn*^-/-^) zebrafish using CRISPR/Cas9 and identified two premature mortality phases: 7–21 and 60–90 days post-fertilization (dpf). Time-course transcriptomics revealed two *wrn*^-/-^ subgroups. One showed the reduced expression of intestinal and pancreatic exocrine genes at 7–9 dpf, while the other maintained normal expression initially but eventually showed reduced pancreatic exocrine genes by 21–35 dpf. The prematurely dying *wrn*^-/-^ larvae exhibited intestinal villi and pancreatic defects, along with DNA damage, cell-cycle arrest, and apoptosis. They also had lower glycogen, glucose, and fat levels compared to wild-type and late-dying *wrn*^-/-^ larvae, suggesting malnutrition. Notably, excess feeding partially improved their survival. These findings reveal early pathological features in the zebrafish model of Werner syndrome.

## Highlights

• Zebrafish *wrn* truncated mutants segregate into mild and severe phenotypic classes
• Severe *wrn* mutants showed high DNA damage and apoptosis, causing gut and pancreatic defects
• Severe *wrn* mutants showed low glucose, glycogen, and fat levels, indicating malnutrition
• Increased food availability partially rescued the survival of *wrn* mutant zebrafish

## Graphical abstract

**Figure:**
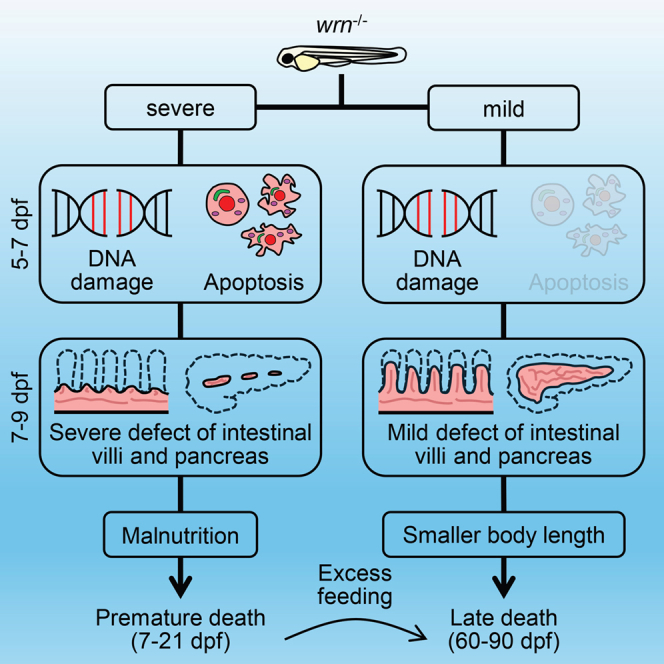

## Introduction

Progeria and related progeroid syndromes are genetic disorders characterized by the premature onset of age-related diseases. Typical examples include Hutchinson-Gilford progeria syndrome,^1^ Bloom syndrome,^2^ Cockayne syndrome,^3^ Xeroderma pigmentosum (XP),^4^ Ataxia-telangiectasia (A-T),^5^ and Werner syndrome.^6^ Hutchinson-Gilford progeria syndrome, Bloom Syndrome, and Cockayne syndrome occur very early (1–10) years of life, whereas Werner syndrome occurs around the age of 20. The symptoms of Werner syndrome include gray hair, cataracts, dermal atrophy, insulin-resistant diabetes, and arteriosclerosis. Individuals with Werner syndrome also exhibit characteristic short stature. Many variants of the Werner syndrome gene *WRN* have been identified in human patients.^7^ The human WRN protein consists of 1,432 amino acids and contains multiple functional domains, such as exonuclease, helicase, RecQ C-terminal (RQC), helicase-and-RNaseD-like-C-terminal (HRDC), and nuclear localization signal (NLS) domains.^8^ It plays critical roles in DNA repair, replication, transcription, and telomere maintenance, and is widely conserved across species from bacteria (*E. coli*) to humans.^7^^,^^9^ However, the pathogenic basis of how *WRN* loss causes accelerated aging in Werner syndrome remains largely unclear, and effective treatments to prevent premature aging have not been established. In addition, early-onset symptoms before the age of 20 have not been thoroughly explored.

In previous cellular studies, mesenchymal stem cells differentiated from *WRN*-null embryonic stem cells exhibited reduced cell proliferative capacity and increased senescence-associated β-galactosidase (SA-β-gal) activity, with the elevated expression of senescent markers such as *p15*^Ink4a^ and *p21*^Waf1^.^10^ Interestingly, while induced pluripotent stem (iPS) cells derived from skin fibroblasts of patients with Werner syndrome were reported to maintain undifferentiated states without the upregulation of senescence-associated genes after two years of long-term culture,^11^ a recent study demonstrated that Werner syndrome iPS cells can exhibit accelerated senescence compared to wild-type cells under certain conditions,^12^ suggesting context-dependent variability in senescence phenotypes. *Wrn*-deficient mouse models have yielded complex results. Mice lacking part of the helicase domain (*Wrn*^Δhel/Δhel^) exhibit increased genomic instability and a modest reduction in lifespan.^13^^,^^14^^,^^15^ In contrast, mice completely lacking *Wrn* (*Wrn*^-/-^) do not show overt progeroid features and live beyond two years without obvious aging-related phenotypes.^16^ However, when *Wrn*^−/−^ is combined with mutations in *p53* or the telomerase RNA component (*Terc*), these double mutants display shortened lifespans and features associated with genomic instability and premature aging.^16^^,^^17^ These findings suggest that *Wrn* loss alone is insufficient to trigger overt progeroid phenotypes in mice, thereby emphasizing the necessity of utilizing alternative model systems to investigate the physiological basis of Werner Syndrome.

We employed zebrafish (*Danio rerio*), an increasingly utilized vertebrate model for studying development, behavior, and disease,^18^ to establish a model for Werner syndrome. Under standard laboratory conditions, zebrafish typically live for 2 to 3 years. During early development, embryos depend on yolk-derived nutrients until approximately 5 days post-fertilization (dpf), at which point they begin external feeding.^19^ The larval stage spans from 5 to around 30 dpf, followed by the juvenile period from 30 to 90 dpf, with sexual maturity reached at approximately 90 dpf.^20^ These well-defined developmental transitions make zebrafish a valuable model for investigating growth, metabolism, and aging in vertebrates. A previous study reported a nonsense mutant allele (*wrn*^sa34829^, Q431∗) of *wrn* in zebrafish that causes truncation prior to the helicase domain.^21^ Mutant embryos exhibited shorter body length at 40 hours post-fertilization (hpf) during embryogenesis and showed the impairment of cartilage and bone development. Another study reported two mutant alleles of *wrn* in zebrafish: a nonsense mutation (*wrn*^cq216^, L173∗) resulting in the truncation of the exonuclease domain, and an internal deletion allele (*wrn*^cq217^) causing a large deletion spanning from the middle of the exonuclease domain to the end of the HRDC domain.^22^ These mutants exhibited increased SA-β-gal activity without apparent morphological defects in intestine at 8 dpf, showed reduced body length at 30 dpf, and died before 60 dpf. Treatment with sapanisertib, an mTOR inhibitor, suppressed SA-β-gal activity in the intestine of the mutant.

Here, we independently generated a zebrafish *wrn* mutant carrying a 2-bp deletion that causes a frameshift in the helicase domain. Unlike the previous study, our *wrn* mutants did not exhibit shortened body length during embryonic stages. Approximately 90% of *wrn* mutant larvae died prematurely by 30 dpf, whereas the remaining 10% showed growth retardation and died by 90 dpf. Transcriptome analysis revealed that the prematurely dying *wrn* mutant larvae exhibited defects in the pancreatic exocrine and intestine cells. Consistent with these findings, we found that *wrn* mutant larvae were malnourished due to impaired digestion and absorption.

## Results

### Zebrafish *wrn* mutants were small at the larval stage and were short-lived

**Figure 1:**
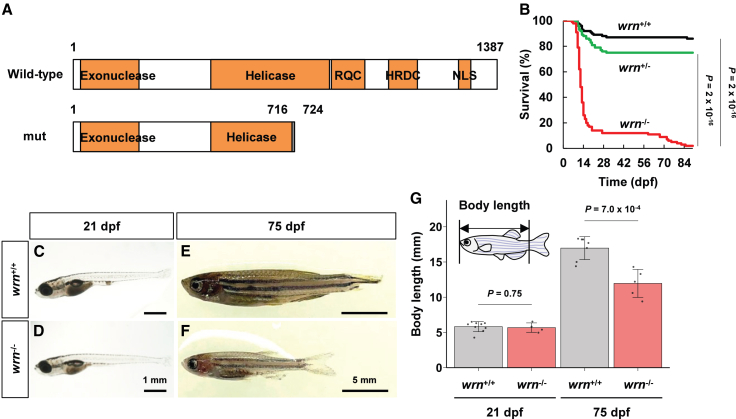
The *wrn*^-/-^ zebrafish exhibit short stature and undergo premature death (A) Domain structures of the wild-type and mutant zebrafish Wrn protein. (Top) Wild-type Wrn protein contains exonuclease, helicase, RecQ C-terminal (RQC), helicase and RNaseD-like C-terminal (HRDC), and nuclear localization signal (NLS) domains. (Bottom) A frameshift mutation results in a truncation within the helicase domain. (B) Survival curves of *wrn*^+/+^ (black, *n* = 214), *wrn*^+/^^-^ (green, *n* = 472) and *wrn*^-/-^ (red, *n* = 133) zebrafish. Statistical significance was assessed using the log-rank test. (C–F) Images of (C) *wrn*^+/+^ (*n* = 9) and (D) *wrn*^-/-^ (*n* = 4) larvae at 21 dpf (scale bars, 1 mm), and (E) *wrn*^+/+^ (*n* = 7) and (F) *wrn*^-/-^ (*n* = 5) juveniles at 75 dpf (scale bars, 5 mm). (G) Body length measurements of *wrn*^+/+^ larvae (*n* = 9) and *wrn*^-/-^ larvae (*n* = 4) at 21 dpf, and *wrn*^+/+^ juveniles (*n* = 7) and *wrn*^-/-^ juveniles (*n* = 5) at 75 dpf. Statistical significance was assessed using an unpaired two-tailed Student’s *t* test. Data are presented as mean ± standard deviation (SD). The detailed mean ± SD are shown in Table S2.

To generate *wrn*^-/-^ zebrafish, we designed a CRISPR that targets *wrn* gene and obtained an allele with a 2-bp deletion in exon 19 (Figures S1A and S1B). This mutant allele harbored a frameshift after the alanine residue at position 716 (T717Sfs∗9) that is located at two-thirds of the helicase domain (Figures 1A and S2). The 2-bp deletion was detectable by PCR, which enabled the genotyping of *wrn*^+/+^, *wrn* ^+/-^, and *wrn*^-/-^ (Figure S1C). The transcription level of *wrn* mRNA in *wrn*^-/-^ was comparable to those in *wrn*^+/+^ and *wrn*^+/^^-^ (Figure S1D, Table S1). To assess whether *wrn* deficiency affects embryogenesis, we first examined the morphology of *wrn*^+/+^ and *wrn*^-/-^ individuals at 1–5 dpf. We did not find any morphological defects in *wrn*^-/-^ embryos/larvae by 5 dpf. Since another *wrn* mutant zebrafish carrying a nonsense mutation (Q431∗) upstream of the helicase domain showed developmental malformations and reduced body length by 2 dpf,^21^ our mutant allele is considered to exhibit milder phenotypes and thus is amenable to post-embryonic analyses. To address whether *wrn* deficiency affects the lifespan of zebrafish, we conducted a survival experiment. The *wrn*^+/+^, *wrn* ^+/-^, and *wrn*^-/-^ larvae obtained from *wrn*^+/-^ crosses were phenotypically indistinguishable at 5 dpf. They were transferred to the water circulation system at 5 dpf and reared up to 90 dpf. Dead larvae/juveniles were collected every day and subjected to genotyping. In total, 86% (185/214) of *wrn*^+/+^ and 75% (353/472) of *wrn*^+/-^ larvae survived for 90 days, whereas 86% (114/133) of *wrn*^-/-^ larvae died at 7–21 dpf (Figure 1B). Although *wrn*^-/-^ larvae that survived beyond 21 dpf appeared healthy and were indistinguishable from *wrn*^+/+^ larvae at that stage (Figures 1C, 1D, and 1G, and Table S2), they eventually became smaller than *wrn*^+/+^ juveniles by 75 dpf and exhibited disrupted pigmentation with irregular stripe patterns (Figures 1E–1G, Table S2). The remaining 14% (19/133) of *wrn*^-/-^ juveniles died between 60 and 90 dpf. These results demonstrate that *wrn*^-/-^ zebrafish showed two different phases of mortality: an early phase at 7–21 dpf, referred to as premature-death, and a subsequent phase at 60–90 dpf, referred to as late-death. These *wrn*^-/-^ phenotypes differ from those reported for previously described *wrn* mutants.^21^^,^^22^^,^^23^

### Decreased expression of intestinal and pancreatic exocrine genes in *wrn*^-/-^ zebrafish

**Figure 2:**
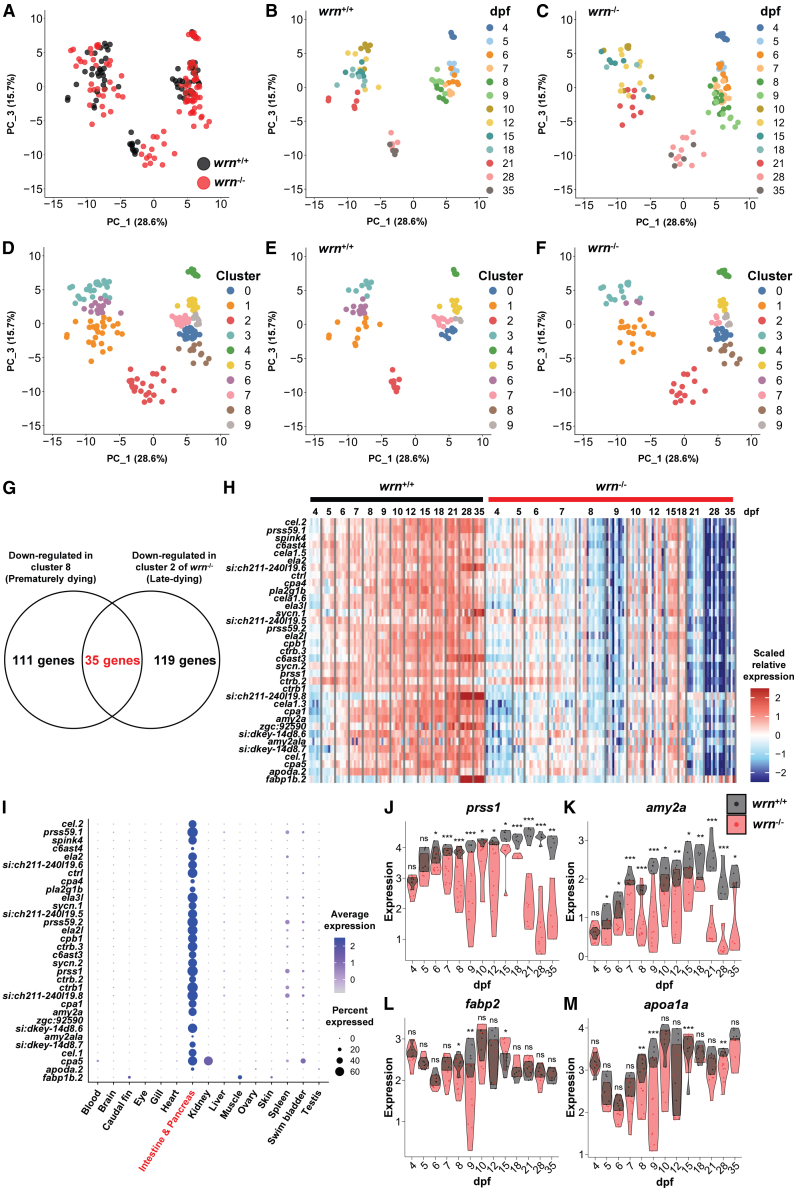
Expression of pancreatic cell and intestinal markers is decreased in zebrafish *wrn*^-/-^ (A) Principal component analysis (PCA) of the *wrn*^+/+^ and *wrn*^-/-^ individuals, color-coded by genotype. (B) PCA of the *wrn*^+/+^ individuals, colored by developmental stage. (C) PCA of the *wrn*^-/-^ individuals, colored by developmental stage. (D) PCA based on clustering, color-coded by cluster identity. (E) PCA of the *wrn*^+/+^ individuals, color-coded by cluster identity from Figure 2D. (F) PCA of the *wrn*^-/-^ individuals, color-coded by cluster identity from Figure 2D. (G) Gene filtering strategy used to identify 35 genes that were specifically downregulated in *wrn*^-/-^. (H) Heatmap of 35 genes downregulated in *wrn*^-/-^ at both 7–9 and 21–35 dpf. (I) Tissue-specific expression of 32 genes (excluding *cela1.3*, *cela1.5,* and *cela1.6*) based on the Zebrafish Cell Landscape. (J–M) Expression of pancreatic exocrine cell and intestinal markers in *wrn*^+/+^ (black) and *wrn*^-/-^ (red). Exocrine cell markers: *prss1* (J) and *amy2a* (K); Intestinal markers: *fabp2* (L) and *apoa1a* (M). Statistical significance was assessed using Welch’s *t* test at each time point. ∗*p* < 0.05, ∗∗*p* < 0.01, ∗∗∗*p* < 0.001, ns: not significant. The detailed *p*-values are shown in Table S5.

To address what occurs in *wrn*^-/-^ larvae when they undergo premature death in larval stages, we employed time-course transcriptome analysis for individual larvae. RNA was extracted from whole zebrafish larvae of *wrn*^+/+^ and *wrn*^-/-^ at 13 different time points that covered the period of premature death (4–35 dpf; *n* = 3–8, each) and subjected to transcriptome analysis (Table S3). Principal component analysis (PCA) showed that *wrn*^+/+^ larvae from 5 to 9 dpf exhibited relatively uniform gene expression profiles, with a notable trajectory transition between 9 and 10 dpf (Figures 2A and 2B). Subsequently, samples from 10 to 21 dpf formed a sparse cluster, followed by another trajectory shift in gene expression between 21 and 28 dpf. The transcriptomic landscape of *wrn*^-/-^ larvae showed a similar distribution of data points to *wrn*^+/+^ up to 6 dpf (Figure 2C). However, starting at 7–9 dpf, a subset of *wrn*^-/-^ samples began to deviate from the *wrn*^+/+^ group, temporally associated with the onset of premature death. Interestingly, this early divergence preceded the appearance of visible morphological abnormalities, implying that transcriptomic changes occurred prior to phenotypic manifestations. Although most *wrn*^-/-^ larvae that survived beyond 9 dpf displayed transcriptomic profiles similar to those of *wrn*^+/+^ larvae at 10–21 dpf, their gene expression patterns began to diverge again from 28 to 35 dpf. Clustering analysis further classified the samples into ten groups (Figures 2D and 2E). Notably, Cluster 8 consisted exclusively of *wrn*^-/-^ larvae from 7 to 9 dpf, likely representing the premature-death population, which undergoes mortality by 21 dpf (Figure 2F). In contrast, both *wrn*^+/+^ and *wrn*^-/-^ larvae at 28–35 dpf were grouped in Cluster 2. The *wrn*^-/-^ larvae in Cluster 2 likely represent the late-death population, which undergoes mortality between 60 and 90 dpf.

We investigated gene expression changes in *wrn*^-/-^ larvae to explore potential causes of lethality. Among the 21,887 differentially expressed genes across all samples, 108 genes were significantly upregulated in Cluster 8 at 7–9 dpf (Figure S3A). But gene ontology (GO) enrichment analysis of these genes yielded no statistically significant terms. In contrast, 146 genes were downregulated in Cluster 8 (Figures 2G, S3B, and S3C). Among these, 35 genes were downregulated in both *wrn*^-/-^ larvae from Cluster 8 and Cluster 2, both of which are associated with increased mortality (Figures 2G, 2H, and S3D, Table S4). Analysis using the online zebrafish single-cell transcriptome atlas revealed that 26 out of the 35 genes are specifically expressed in pancreatic exocrine and epsilon cells, as well as in intestinal enterocytes and goblet cells (Figures 2I and S3E). Expression of pancreatic exocrine markers *prss1* and *amy2a* was indeed reduced in *wrn*^-/-^ larvae at both 7–9 and 21–35 dpf (Figures 2J and 2K; Table S5), and intestinal markers *fabp2* and *apoa1a* were downregulated at 8–9 dpf (Figures 2L and 2M, Table S5). These results suggest that the pathological features of *wrn*^-/-^ lethality are attributable to the impaired pancreatic exocrine and intestinal function.

We also evaluated the temporal progression of DNA damage and cellular senescence. The expression of the DNA damage marker *p53* was increased in *wrn*^-/-^ larvae beginning at 7 dpf and gradually intensified thereafter (Figure S3F, Table S5). In contrast, the expression levels of the cellular senescence markers *p21* and *p16* did not differ between *wrn*^+/+^ and *wrn*^-/-^ larvae up to 35 dpf (Figures S3G and S3H, Table S5). These results suggest that DNA damage occurs in *wrn*^-/-^ larvae as early as 7 dpf, whereas cellular senescence does not progress at least until 35 dpf.

### Progressive histological defects in the intestine and pancreas of *wrn*^-/-^ larvae during the premature death stage

**Figure 3:**
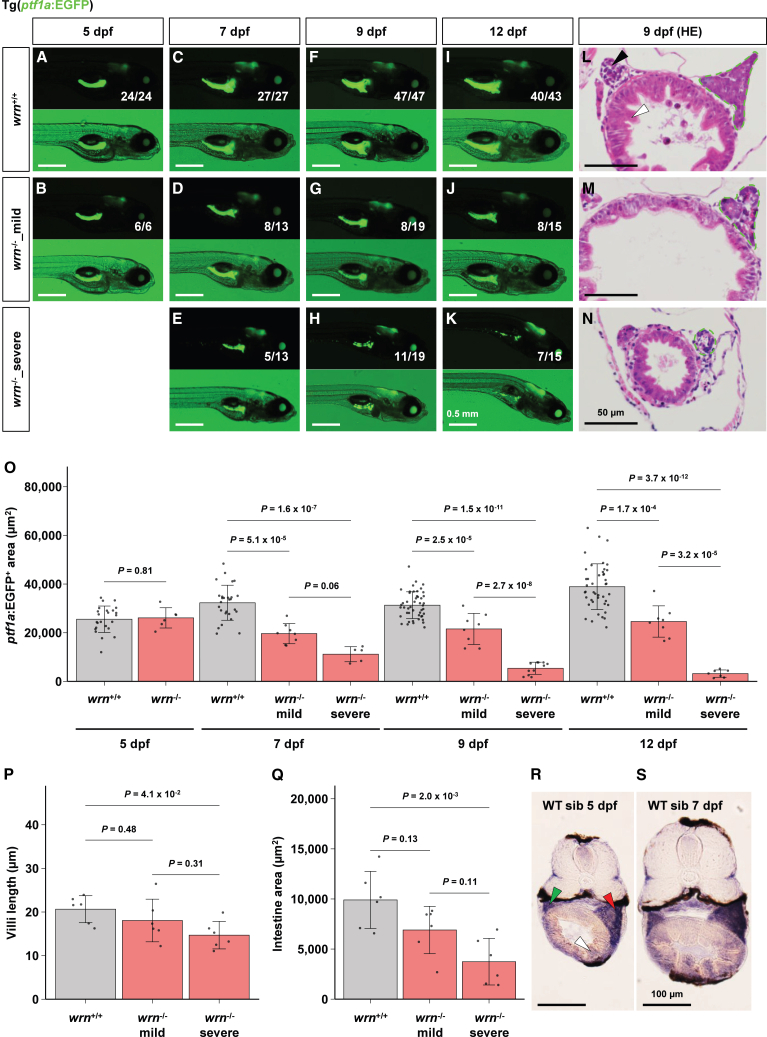
Morphological abnormalities of pancreas and intestine in *wrn*^-/-^ larvae (A–K) Morphology of pancreatic exocrine cell in Tg(*ptf1a:EGFP*); *casper* larvae. (A) The *wrn*^+/+^ (*n* = 24/24) and (B) *wrn*^-/-^ (*n* = 6/6) larvae at 5 dpf. (C) The *wrn*^+/+^ (*n* = 27/27), (D) *wrn*^-/-^_mild (*n* = 8/13), and (E) *wrn*^-/-^_severe (*n* = 5/13) larvae at 7 dpf. (F) The *wrn*^+/+^ (*n* = 47/47), (G) *wrn*^-/-^_mild (*n* = 8/19), and (H) *wrn*^-/-^_severe (*n* = 11/19) larvae at 9 dpf. (I) The *wrn*^+/+^ (*n* = 40/43), (J) *wrn*^-/-^_mild (*n* = 8/15), and (K) *wrn*^-/-^_severe (*n* = 7/15) larvae at 12 dpf. Scale bars, 0.5 mm. (L–N) HE staining of a paraffin section. (L) The *wrn*^+/+^ (*n* = 6), (M) *wrn*^-/-^_mild (*n* = 6), and (N) *wrn*^-/-^_severe (*n* = 6) larvae at 9 dpf. Dashed green boxes indicate the pancreatic exocrine region. Black and white arrowheads indicate the liver and intestine, respectively. Scale bars, 50 μm. (O) Area of GFP-positive pancreatic exocrine tissue at 5, 7, 9, and 12 dpf (corresponding to Figures 3A–3K). The number of samples. *wrn*^+/+^ (*n* = 24): *wrn*^-/-^ (*n* = 6) at 5 dpf; *wrn*^+/+^ (*n* = 27): *wrn*^-/-^_mild (*n* = 8): *wrn*^-/-^_severe (*n* = 5) at 7 dpf; *wrn*^+/+^ (*n* = 47): *wrn*^-/-^_mild (*n* = 8): *wrn*^-/-^_severe (*n* = 11) at 9 dpf; *wrn*^+/+^ (*n* = 43): *wrn*^-/-^_mild (*n* = 8): *wrn*^-/-^_severe (*n* = 7) at 12 dpf. Statistical significance at 5 dpf was assessed using an unpaired two-tailed Student’s *t* test, and that at 7, 9, and 12 dpf was assessed using one-way ANOVA followed by Tukey’s honest significant difference (HSD) test. Data are presented as mean ± SD. The detailed mean ± SD are shown in Table S6. (P and Q) Quantification of average intestinal villi length (P) and intestine area (Q) of *wrn*^+/+^ (*n* = 6), *wrn*^-/-^_mild (*n* = 6), and *wrn*^-/-^_severe (*n* = 6) at 9 dpf (corresponding to Figures 3L–3N). Statistical significance was assessed using one-way ANOVA followed by Tukey’s HSD test. Data are presented as mean ± SD. The detailed mean ± SD are shown in Table S7. (R and S) *In situ* hybridization of *wrn* expression in wild-type sibling (WT sib) zebrafish at 5 dpf (R) and 7 dpf (S). Red, white, and green arrowheads indicate the pancreas, intestine, and liver, respectively. Scale bars, 100 μm.

To examine the histological abnormalities in *wrn*^-/-^ larvae, we used a transgenic zebrafish line Tg(*ptf1a:EGFP*), which labels pancreatic exocrine cells and thus reveals the overall shape of the pancreas.^24^^,^^25^ At 5 dpf, GFP-positive exocrine cells were observed beneath the swim bladder on the right side of the body in both *wrn*^+/+^ and *wrn*^-/-^ larvae (Figures 3A, 3B, and 3O; Table S6). By 7 dpf, *wrn*^-/-^ larvae could be categorized into two distinct phenotypic groups. One group, hereafter referred to as *wrn*^-/-^_mild, appeared morphologically normal but exhibited a moderately reduced GFP signal (Figures 3C, 3D, and 3O, and Table S6). The other group, referred to as *wrn*^-/-^_severe, appeared emaciated and showed a significantly diminished GFP-positive area, often accompanied by discontinuities and fragmentation (Figures 3E and 3O, and Table S6). At both 9 and 12 dpf, *wrn*^-/-^_mild larvae exhibited smaller GFP-positive exocrine cells compared to *wrn*^+/+^ larvae, while *wrn*^-/-^_severe larvae showed sparse and discontinuous GFP expression (Figures 3F–3K and 3O, and Table S6). These severely affected larvae died within five days of becoming visibly emaciated. These findings suggest that after 5 dpf, a subset of *wrn*^-/-^ larvae stochastically develops pancreatic exocrine defects, leading to progressive emaciation and premature death. Hematoxylin and eosin staining of paraffin sections at 9 dpf confirmed that the pancreas of *wrn*^-/-^_mild larvae was slightly smaller than that of *wrn*^+/+^ larvae, whereas the pancreas of *wrn*^-/-^_severe larvae was markedly smaller than those of *wrn*^+/+^ and *wrn*^-/-^_mild larvae (Figures 3L–3N). The intestinal epithelium of zebrafish contains protruding structures known as villi, which enhance nutrient absorption.^26^^,^^27^ Intestinal villi extending into the gut lumen were observed in *wrn*^+/+^ larvae but appeared shortened in both *wrn*^-/-^_mild and *wrn*^-/-^_severe larvae (Figures 3L–3N and 3P, and Table S7). In addition, the intestinal surface area was reduced in *wrn*^-/-^_mild and *wrn*^-/-^_severe larvae than *wrn*^+/+^ larvae (Figures 3L–3N and 3Q, and Table S7). These histological defects in the pancreas and intestine were consistent with transcriptomic abnormalities. *In situ* hybridization confirmed that *wrn* is expressed in the pancreas and intestine at 5 and 7 dpf larvae (Figures 3R and 3S), supporting the notion that these digestive organs are primarily affected by the loss of *wrn* function.

**Figure 4:**
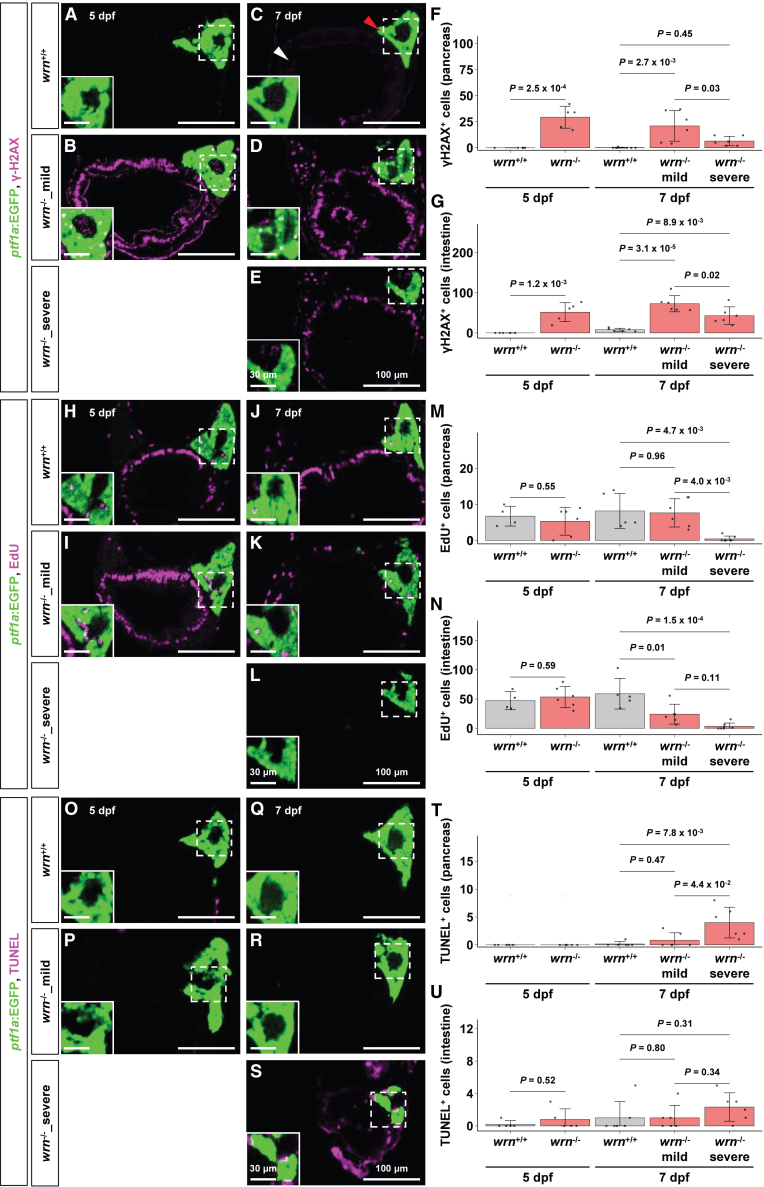
DNA damage, cell death, and proliferation arrest occur in the pancreas and intestine of *wrn*^-/-^ larvae (A–E) gammaH2AX staining of cryosection. (A) The *wrn*^+/+^ (*n* = 5) and (B) *wrn*^-/-^ (*n* = 5) larvae at 5 dpf, and (C) *wrn*^+/+^ (*n* = 6), (D) *wrn*^-/-^_mild (*n* = 6), and (E) *wrn*^-/-^_severe (*n* = 6) larvae at 7 dpf. (F and G) Quantification of gammaH2AX staining signals in pancreas (F) and intestine (G) (corresponding to Figures 4A–4E). The number of samples. *wrn*^+/+^ (*n* = 5): *wrn*^-/-^ (*n* = 5) at 5 dpf; *wrn*^+/+^ (*n* = 6): *wrn*^-/-^_mild (*n* = 6): *wrn*^-/-^_severe (*n* = 6) at 7 dpf. Statistical significance at 5 dpf was assessed using an unpaired two-tailed Student’s *t* test, and that at 7 dpf was assessed using one-way ANOVA followed by Tukey’s HSD test. Data are presented as mean ± SD. The detailed mean ± SD are shown in Table S8. (H–L) EdU labeling of cryosection. (H) The *wrn*^+/+^ (*n* = 4) and (I) *wrn*^-/-^ (*n* = 6) larvae at 5 dpf, and (J) *wrn*^+/+^ (*n* = 5), (K) *wrn*^-/-^_mild (*n* = 6) and (L) *wrn*^-/-^_severe (*n* = 7) larvae at 7 dpf. (M and N) Quantification of EdU-labeled signals in pancreas (M) and intestine (N) (corresponding to Figures 4H–4L). The number of samples. *wrn*^+/+^ (*n* = 4): *wrn*^-/-^ (*n* = 6) at 5 dpf; *wrn*^+/+^ (*n* = 5): *wrn*^-/-^_mild (*n* = 6): *wrn*^-/-^_severe (*n* = 7) at 7 dpf. Statistical significance at 5 dpf was assessed using an unpaired two-tailed Student’s *t* test, and that at 7 dpf was assessed using one-way ANOVA followed by Tukey’s HSD test. Data are presented as mean ± SD. The detailed mean ± SD are shown in Table S8. (O–S) TUNEL labeling of cryosection. (O) The *wrn*^+/+^ (*n* = 5) and (P) *wrn*^-/-^ (*n* = 5) larvae at 5 dpf, and (Q) *wrn*^+/+^ (*n* = 6), (R) *wrn*^-/-^_mild (*n* = 6), and (S) *wrn*^-/-^_severe (*n* = 6) larvae at 7 dpf. (T and U) Quantification of TUNEL labeled signals in pancreas (T) and intestine (U) (corresponding to Figures 4O–4S). The number of samples. *wrn*^+/+^ (*n* = 5): *wrn*^-/-^ (*n* = 5) at 5 dpf; *wrn*^+/+^ (*n* = 6): *wrn*^-/-^_mild (*n* = 6): *wrn*^-/-^_severe (*n* = 6) at 7 dpf. Statistical significance at 5 dpf was assessed using the Wilcoxon rank-sum test, and that at 7 dpf was assessed using the Kruskal-Wallis test followed by Dunn’s multiple comparisons test with Holm correction. Statistical analysis for the pancreas at 5 dpf was not performed because both *wrn*^+/+^ and *wrn*^-/-^ showed zero values. Data are presented as mean ± SD. The detailed mean ± SD are shown in Table S8. (A–E, H–L, and O–S) Scale bars, 100 μm. Scale bars in white box, 30 μm.

Given that WRN protein functions as a DNA helicase involved in DNA damage repair, we hypothesized that *wrn*^-/-^ larvae fail to repair DNA damage, resulting in cell-cycle arrest and apoptosis in the pancreas and intestine. At 5 dpf, γ-H2AX immunostaining of cryosections from Tg(*ptf1a:EGFP*) larvae showed no signal of DNA damage in the intestine or GFP-labeled pancreas of *wrn*^+/+^ larvae (Figure 4A). In contrast, γ-H2AX signals were shown in both tissues of *wrn*^-/-^ larvae (Figures 4B, 4F, and 4G, and Table S8). At 7 dpf, γ-H2AX signals were not detected in either tissue of *wrn*^+/+^ larvae (Figure 4C), whereas clear signals were observed in both *wrn*^-/-^_mild and *wrn*^-/-^_severe larvae (Figures 4D–4G, Table S8). Interestingly, the signal intensity in *wrn*^-/-^_severe larvae was reduced in both tissues (Figures 4F and 4G, Table S8). EdU labeling revealed the presence of proliferating cells in both the intestine and pancreas of *wrn*^+/+^ and *wrn*^-/-^ larvae at 5 dpf (Figures 4H, 4I, 4M, and 4N, and Table S8). At 7 dpf, EdU-positive cells remained detectable in the pancreas of *wrn*^+/+^ and *wrn*^-/-^_mild larvae, but were absent in *wrn*^-/-^_severe larvae (Figures 4J–4M, Table S8). In the intestine, the number of EdU-positive cells was significantly reduced in both *wrn*^-/-^_mild and *wrn*^-/-^_severe larvae compared to *wrn*^+/+^ larvae (Figures 4J–4L and 4N, and Table S8). TUNEL labeling showed little or no cell death in either the pancreas or intestine of *wrn*^+/+^ and *wrn*^-/-^ larvae at 5 dpf (Figures 4O, 4P, 4T, and 4U, and Table S8). At 7 dpf, TUNEL-positive cells showed little in both pancreas and intestine of *wrn*^+/+^ and *wrn*^-/-^_mild larvae (Figures 4Q, 4R, 4T, and 4U, and Table S8). In contrast, these cells were shown in both tissues of *wrn*^-/-^_severe larvae, appearing prominently in the pancreas and to an extent in the intestine (Figures 4S–4U, Table S8). Taken together, these findings suggest that *wrn* deficiency leads to DNA damage, resulting in impaired cell proliferation and increased apoptosis in digestive tissues during the premature death stage.

A previous study reported that telomere length in the intestine of *wrn*-deficient zebrafish was shorter than that of wild-type fish.^22^ In our study, we measured whole-body telomere length using a qPCR-based method and found no significant shortening in *wrn*^-/-^_severe larvae compared to *wrn*^+/+^ or *wrn*^-/-^_mild larvae at 7 dpf (Figure S4, Table S9). These findings suggest that the observed abnormalities in the pancreas and intestine are more likely attributable to the accumulation of DNA damage rather than telomere shortening at this developmental stage.

### The *wrn*^-/-^ zebrafish were malnourished

**Figure 5:**
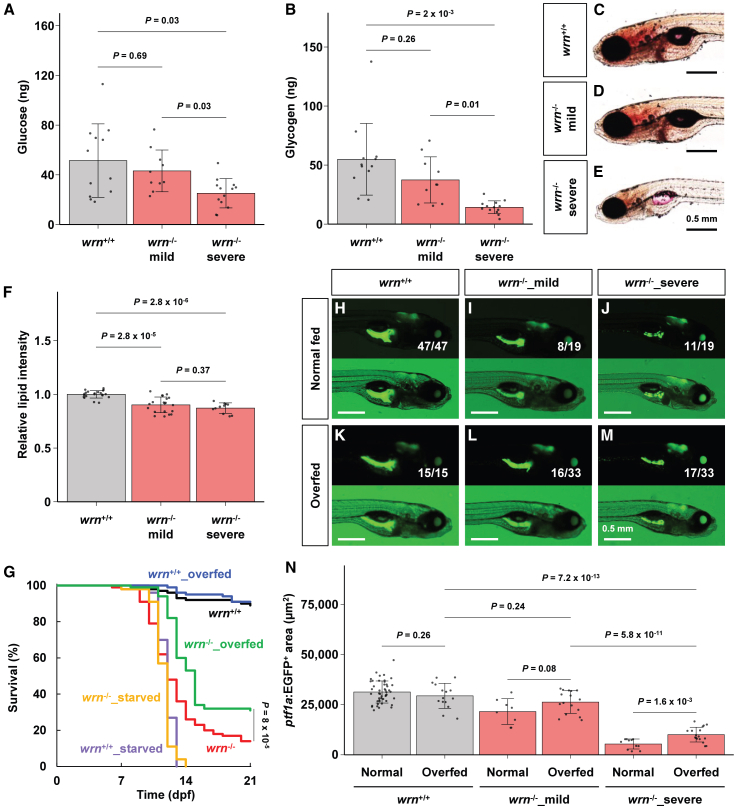
Prematurely dying *wrn*^-/-^ zebrafish become malnourished (A and B) (A) Glucose and (B) glycogen levels in whole larvae at 9 dpf. The number of samples. *wrn*^+/+^ (*n* = 12); *wrn*^-/-^_mild (*n* = 10); *wrn*^-/-^_severe (*n* = 13). Statistical significance was assessed using Welch’s ANOVA followed by Games-Howell post-hoc test. Data are presented as mean ± SD. The detailed mean ± SD are shown in Table S10. (C–E) Oil Red O staining of whole-mount larvae. (C) The *wrn*^+/+^ (*n* = 21), (D) *wrn*^-/-^_mild (*n* = 20), and (E) *wrn*^-/-^_severe (*n* = 11) larvae at 9 dpf. Scale bars, 0.5 mm. (F) Quantification of Oil Red O signal intensity (corresponding to Figures 5C–5E). The number of samples. *wrn*^+/+^ (*n* = 21): *wrn*^-/-^_mild (*n* = 20): *wrn*^-/-^_severe (*n* = 11). Statistical significance was assessed using Welch’s ANOVA followed by Games-Howell post-hoc test. Data are presented as mean ± SD. The detailed mean ± SD are shown in Table S11. (G) Survival assay under normal, starved, and overfed conditions. The *wrn*^+/+^_starved (purple, *n* = 56) and *wrn*^-/-^_starved (orange, *n* = 54); the *wrn*^+/+^_overfed (blue, *n* = 78) and *wrn*^-/-^_overfed (green, *n* = 68). The *wrn*^+/+^ (black, *n* = 214) and *wrn*^-/-^ (red, *n* = 133) under normal conditions (data reused from Figure 1B). Statistical significance was assessed using the log-rank test. (H–M) Morphology of pancreatic exocrine cell in Tg(*ptf1a:EGFP*); *casper* larvae at 9 dpf. (H) The *wrn*^+/+^ (*n* = 47/47), (I) *wrn*^-/-^_mild (*n* = 8/19), and (J) *wrn*^-/-^_severe (*n* = 11/19) larvae under normal feed conditions (data reused from Figures 3F–3H). (K) The *wrn*^+/+^ (*n* = 15/15), (L) *wrn*^-/-^_mild (*n* = 16/33), and (M) *wrn*^-/-^_severe (*n* = 17/33) larvae under overfeed condition. Scale bars, 0.5 mm. (N) Area of GFP-positive pancreatic exocrine tissue at 9 dpf (corresponding to Figures 5H–5M). The number of samples. *wrn*^+/+^ (*n* = 47): *wrn*^-/-^_mild (*n* = 8): *wrn*^-/-^_severe (*n* = 11) under normal feed condition; *wrn*^+/+^ (*n* = 15): *wrn*^-/-^_mild (*n* = 16): *wrn*^-/-^_severe (*n* = 17) under overfeed condition. Statistical significance was assessed using an unpaired two-tailed Student’s *t* test for comparisons between conditions (e.g., *wrn*^+/+^ under normal conditions vs. *wrn*^+/+^ under overfeed condition), and one-way ANOVA followed by Tukey’s HSD test for comparisons under overfeed condition (*wrn*^+/+^, *wrn*^-/-^_mild, and *wrn*^-/-^_severe). Data are presented as mean ± SD. The detailed mean ± SD are shown in Table S6.

Since the pancreas and intestine are essential for nutrient digestion and absorption, respectively,^28^^,^^29^ we investigated whether *wrn*^-/-^ larvae exhibit nutritional deficits. We generated larvae by crossing Tg(*ptf1a:EGFP*); *wrn*^+/-^ fish and categorized them as *wrn*^-/-^_mild, *wrn*^-/-^_severe, or phenotypically normal larvae based on GFP signal intensity. Whole larval extracts were collected from 9 dpf larvae for glucose and glycogen quantification, followed by genotyping to confirm their genotypic classifications. Glucose levels in *wrn*^-/-^_severe larvae were significantly lower than those of *wrn*^+/+^ and *wrn*^-/-^_mild larvae (Figure 5A, Table S10). Similarly, glycogen levels were markedly reduced in *wrn*^-/-^_severe larvae compared with *wrn*^+/+^ and *wrn*^-/-^_mild larvae (Figure 5B, Table S10). Oil Red O staining indicated that both *wrn*^-/-^_mild and *wrn*^-/-^_severe larvae exhibited lower fat accumulation than *wrn*^+/+^ larvae at 9 dpf (Figures 5C–5F, Table S11). Our transcriptome analysis revealed no significant changes in the expression of genes involved in glycolysis (*hk1*), gluconeogenesis (*pcca*), tricarboxylic acid cycle (*cs*), or fatty acid metabolism (*acadm*) in *wrn*^-/-^ larvae (Figures S5A–S5D, Table S5). In contrast, genes associated with autophagy, such as *atg9a*, *atg14*, *optn,* and *sqstm1*, which are typically upregulated during starvation,^30^^,^^31^^,^^32^^,^^33^ were elevated in *wrn*^-/-^ larvae compared to *wrn*^+/+^ at 9 dpf, consistent with a starvation-like response (Figures S5E–S5H, Table S5). Furthermore, *atg9* and *optn* are also implicated in mitophagy,^34^ and both *optn* and *sqstm1* are known to be upregulated in response to oxidative stress.^35^ Expression levels of mitophagy markers *bnip3lb* and *fis1*^36^^,^^37^ were elevated in *wrn*^-/-^ larvae compared to *wrn*^+/+^ at 9 dpf (Figures S5I and 5J, Table S5). Similarly, oxidative stress markers *gclm* and *txn* were also upregulated in *wrn*^-/-^ larvae at same time point (Figures S5K and S5L, Table S5). These findings suggest that *wrn*^-/-^_severe larvae experience malnutrition due to impaired digestive function after 5 dpf, when yolk reserves are depleted. This nutrient deficiency likely induces autophagy, which subsequently triggers mitophagy and enhances oxidative stress, leading to progressive cellular damage in the pancreas and intestine of *wrn*^-/-^_severe larvae.

If *wrn*^-/-^_severe larvae die from malnutrition after 5 dpf, providing excess food might improve their survival by facilitating easier access to nutrients. To test this hypothesis, we repeated the survival assay using larvae from *wrn*^+/-^ crosses and raised them either with excess food or without food. When *wrn*^+/+^ and *wrn*^-/-^ larvae were raised without food, all individuals died between 9 and 14 dpf regardless of genotype, coinciding with the period during which *wrn*^-/-^_severe larvae undergo premature death (Figure 5G). Under excess feeding, *wrn*^+/+^ larvae showed no improvement in survival compared with control-fed larvae, whereas the survival of *wrn*^-/-^ larvae improved by approximately 20% by 21 dpf. To further investigate the rescue effects of overfeeding in *wrn*^-/-^ larvae, we classified control-fed and overfed *wrn*^-/-^ larvae into *wrn*^-/-^_mild and *wrn*^-/-^_severe groups based on the morphology of the pancreatic exocrine tissue (Figures 5H–5M). Compared with control-fed larvae, overfeeding increased the size of the pancreas in *wrn*^-/-^_severe larvae (Figure 5N, Table S6). These results suggest that malnutrition is the primary cause of premature death in *wrn*^-/-^ larvae and that nutritional supplementation can extend their survival by promoting recovery of pancreatic condition.

## Discussion

**Figure 6:**
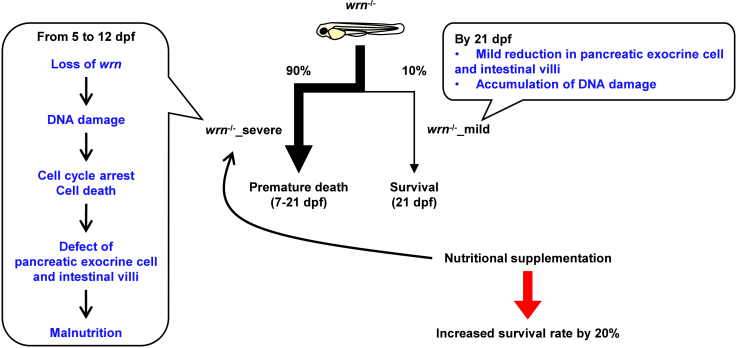
Schematic diagram of *wrn*^-/-^ zebrafish Approximately 90% of *wrn*^-/-^ larvae exhibited defects in pancreas and intestine and underwent premature death between 7 and 21 dpf as a result of pancreas and intestine defects and subsequent malnutrition. Nutritional supplementation increased their survival rate.

The *wrn*-deficient zebrafish exhibited two mortality phases: 90% (*wrn*^-/-^_severe) died prematurely between 7 and 21 dpf, while 10% (*wrn*^-/-^_mild) died later between 60 and 90 dpf (Figure 6). We assume that the emergence of both premature and late-onset mortality results from stochastic variation in the mutant phenotype rather than from additional mutations, because we have maintained our zebrafish *wrn* mutant allele by repeated outcrossing to a wild-type strain, and the occurrence of these two distinct subpopulations has remained consistent across multiple generations. The *wrn*^-/-^_severe larvae displayed DNA damage, reduced cell proliferation, and increased cell death in the intestine and pancreatic exocrine cells. As these tissues are critical for nutrient digestion and absorption, their dysfunction led to malnutrition and early death, which was partially rescued by excess feeding.

### Employment of RNA-Seq

We developed a high-throughput RNA collection protocol^38^ and conducted time-course RNA-Seq in *wrn*^+/+^ and *wrn*^-/-^ larvae. This revealed the downregulation of pancreatic and intestinal genes in *wrn*^-/-^ individuals, identifying digestive tissue defects as a cause of premature death. While we attempted to identify early markers distinguishing *wrn*^-/-^_mild from *wrn*^-/-^_severe, no definitive candidates were found, likely due to limited cell resolution in single-larva RNA samples. Time-course single-cell RNA-Seq may help identify cell-type-specific vulnerabilities or cues preceding the phenotype.

### Autophagy-mitophagy-oxidative stress axis

Our RNA-Seq analysis revealed the upregulation of genes involved in autophagy, mitophagy, and oxidative stress responses in *wrn*^-/-^ zebrafish. This finding is consistent with previous studies showing that WRN deficiency in human cells leads to mitochondrial dysfunction, NAD^+^ depletion, and increased oxidative stress.^12^ Furthermore, NAD^+^ supplementation restores mitophagy and alleviates senescence in WRN-deficient cells.^12^ In our zebrafish model, the elevated expression of autophagy- and mitophagy-related genes (*atg9a*, *optn*, *bnip3lb,* and *fis1*) may reflect a compensatory response to increased oxidative stress and mitochondrial damage. These transcriptomic changes support the notion that metabolic and mitochondrial dysfunction contribute to tissue degeneration in WRN deficiency. Future studies employing functional assays, such as the measurement of ROS, NAD^+^/NADH ratios, and mitophagy flux, will be essential to determine whether these transcriptional changes translate into physiological defects in our model.

### Malnutrition-induced fragility in zebrafish

To determine whether malnutrition contributes to premature death in *wrn*^-/-^ larvae, we performed survival assays under various feeding conditions. Under starvation, both *wrn*^+/+^ and *wrn*^-/-^ larvae died within a similar period (9–14 dpf), suggesting that food intake enhances survival in both genotypes. Overfeeding improved the survival of *wrn*^-/-^ larvae and increased the proportion of individuals exhibiting normal levels of fluorescence in pancreatic exocrine cells, indicating that dietary conditions underlie the early lethality observed in *wrn*^-/-^ larvae. Thus, increased nutrient availability can mitigate mortality in zebrafish. Although exocrine pancreatic insufficiency has not been reported as a common clinical feature of Werner syndrome, short stature, which is a frequent symptom of the disease, may be partly attributable to insufficient nutrient availability. Indeed, a case of Werner syndrome accompanied by malnutrition showed marked improvement after nutritional and vitamin supplementation.^39^

The apparent discrepancy between the beneficial effects of overfeeding and those of mTOR inhibition^22^ may be explained by differences in the underlying mechanisms. Overfeeding likely alleviates the secondary effects of malnutrition by improving nutrient accessibility, thereby supporting overall metabolism and organ maintenance in *wrn*^-/-^ larvae. In contrast, mTOR inhibitors act directly on the nutrient-sensing pathway to reduce the hyperactivation of mTOR signaling, which has been implicated in the pathogenesis of *wrn* deficiency. Thus, although both interventions improve survival, they may act through distinct mechanisms: overfeeding compensating for nutritional deficiency at the organismal level, and mTOR inhibition mitigating cellular stress at the molecular level.

### Phenotypic variability depending on mutation types

Our zebrafish *wrn* mutant (*wrn*^agu26^) exhibits a stronger phenotype than the *wrn* mutants reported (*wrn*^cq216^ and *wrn*^cq217^) by Ma et al., despite retaining a greater portion of the protein.^22^ One possible explanation is that the truncated protein in our mutant retains the intact N-terminal exonuclease domain, which may exhibit altered biochemical properties or aberrant interactions, rather than producing typical WRN functions. In addition, residual domains in the truncated protein may misfold or mislocalize, further exacerbating the phenotype. Differences in the timing of developmental analysis, tissue-specific sensitivity, and the assays used to evaluate phenotypes may also contribute to the apparent discrepancy. Interestingly, similar observations have been reported in mouse models of Werner syndrome. Complete loss of *Wrn* function produces relatively mild phenotypes, whereas mutations that retain partial domains can result in more pronounced abnormalities, likely due to misfolded or misprocessed truncated proteins that perturb cellular homeostasis.^14^^,^^16^^,^^40^^,^^41^ These facts suggest that the presence of a truncated, partially functional WRN protein can sometimes be more deleterious than complete loss of the protein, consistent with the stronger phenotypes observed in our zebrafish mutant. Thus, both in zebrafish and mice, the type of mutation and the domains retained critically influence phenotypic severity, highlighting the importance of mutation-specific effects in WRN deficiency models. Individuals with Werner syndrome harboring WRN gene mutations also exhibit diverse clinical features that do not correlate closely with the extent of WRN protein retained.^6^

### Potential compensatory roles of other RecQ helicases in *wrn*-deficient zebrafish

Previous studies have shown that RecQ helicases perform overlapping and compensatory functions in maintaining genomic stability.^42^^,^^43^ In human cells, RECQL5 cooperates with WRN to ensure proper DNA replication and repair, and simultaneous loss of both helicases results in synergistic defects in replication fork recovery and genome maintenance.^44^ Similarly, BLM and WRN have been reported to function together in the resolution of stalled replication forks and homologous recombination intermediates.^45^^,^^46^ RECQL4 also shares partially redundant roles with WRN in DNA repair processes.^47^ These findings suggest that other RecQ helicases, such as *recql5*, *blm*, and *recql4*, may compensate for *wrn* deficiency in zebrafish. The variable phenotypic severity observed among *wrn*^-/-^ larvae could therefore reflect differences in the degree of such compensatory responses. To examine this possibility, generating double mutants lacking *wrn* and another RecQ helicase, such as *recql5* or *blm,* would be informative. Comparative analyses of their survival, growth, and genomic stability could reveal whether these genes mitigate the effects of WRN loss *in vivo*. Such investigations would deepen our understanding of the functional redundancy within the RecQ helicase family and clarify the molecular basis underlying phenotypic variability in Werner syndrome.

### Potential of late-dying *wrn*^-/-^ zebrafish as Werner syndrome model

In humans, *WRN* mutations cause Werner syndrome, characterized by premature aging symptoms by age 20. In contrast, mice with *WRN* deficiency alone cause mild effects in progeroid features unless combined with *p53* or telomerase mutations.^13^^,^^14^^,^^16^^,^^17^^,^^48^ On the other hand, *wrn*-deficient zebrafish display phenotypes with single-gene disruption. Some (*wrn*^-/-^_severe) exhibit malnutrition due to intestinal and pancreatic defects during larval stages, while others (*wrn*^-/-^_mild) show growth retardation and reduced lifespan later. In humans, infertility is one of the major clinical features of Werner syndrome.^6^ However, we were unable to assess whether our *wrn*^-/-^ zebrafish reached sexual maturity, as they died around 90 dpf, which coincides with the typical onset of sexual maturation in zebrafish. In the present study, we mainly focused on the larval stages of *wrn*^-/-^ zebrafish to define their baseline phenotypes. However, a subset of mutants exhibited late mortality, which may correspond to adult-onset manifestations observed in human Werner syndrome. This late-death phase could provide an important window for investigating progressive aspects of the disease, including cellular senescence, metabolic impairment, and oxidative stress accumulation. Future studies focusing on these later stages, with assessments of molecular aging markers and mitochondrial function, will help elucidate the mechanisms underlying premature aging and further validate *wrn*^-/-^ zebrafish as a comprehensive model for adult-onset Werner syndrome.

### Limitations of the study

While our study demonstrates that *wrn*^-/-^ zebrafish exhibit Werner syndrome-like phenotypes, several limitations should be acknowledged. First, physiological differences between zebrafish and mammals, including aspects of immune and endocrine regulation, may limit the direct translation of our findings to humans. Second, although bulk RNA-seq provided valuable insights into global transcriptional alterations, it lacks cellular resolution and may obscure tissue-specific or cell-type-specific effects. Considering that some of the observed defects, such as growth retardation and digestive tissue defects, are likely tissue- and cell type-dependent, future studies employing single-cell RNA-Seq or spatial transcriptomics will be crucial for dissecting the heterogeneous cellular responses associated with *wrn* loss. Recent advances in zebrafish single-cell and spatial transcriptomics have successfully mapped tissue- and cell-type-specific expression profiles across developmental and adult stages. Integrating such approaches will enable us to delineate the molecular pathways driving tissue-specific degeneration and identify cell populations most vulnerable to *wrn*^-/-^. Third, we inferred functional changes primarily from transcript levels rather than protein abundance or activity. Future studies combining single-cell or spatial transcriptomics with proteomic or metabolomic profiling will provide a more comprehensive and mechanistic understanding of the role of *wrn* in vertebrate aging and its relevance to human Werner syndrome.

## Resource availability

### Lead contact

Further information and requests for resources should be directed to and will be fulfilled by the lead contact, Hiromi Hirata (hihirata@chem.aoyama.ac.jp).

### Materials availability

Zebrafish *wrn* mutants (*wrn*^agu26^) generated in this study are available from the National BioResource Project (NBRP), Japan, upon request.

### Data and code availability

• RNA-sequencing and quantification data generated in this study have been deposited in the Gene Expression Omnibus (GEO) under accession number GSE276188 and are publicly available.
• This study did not generate custom code.
• All other data and materials supporting the findings of this study are available from the corresponding author upon request.

## Acknowledgments

We thank the members of the Hirata Lab for zebrafish care. We also thank Drs. Kazutoyo Ogino and Shin-Ichi Fukuoka (Aoyama Gakuin University) for their initial research contributions and critical discussions, respectively. This work was supported by the 10.13039/501100025019Support for Pioneering Research Initiated by the Next Generation (SPRING; JPMJSP2103) program of the 10.13039/501100002241Japan Science and Technology Agency (JST) and the 10.13039/100018048Sasakawa Scientific Research Grant (2022-4093) from the 10.13039/501100007807Japan Science Society to K.U.; by the Grant-in-Aid for Transformative Research Areas (24H01726) and the Grant-in-Aid for Scientific Research (C) (23K04681) from the 10.13039/501100001691Japan Society for the Promotion of Science (JSPS) to A.S.; and by the Grant-in-Aid for Scientific Research (C) (25K09827) from JSPS, the Research on Regulatory Harmonization and Evaluation of Pharmaceuticals, Medical Devices, Regenerative and Cellular Therapy Products, Gene Therapy Products, and Cosmetics from the 10.13039/100009619Japan Agency for Medical Research and Development (AMED; 25mk0121308h0301 and 25mk0121318h0001), and the Long-Range Research Initiatives (23-1-03) from the Japan Chemical Industry Association to H.H.

## Author contributions

K.U. conducted and analyzed the majority of the experiments. M. Kataoka generated the *wrn* truncation mutant zebrafish. K.U. and M. Kashima analyzed the transcriptome data. K.U. and H.M. performed transgenic zebrafish analysis. I.K. performed H&E staining. K.U., R.S., M.O., and A.S. measured glucose and glycogen levels. K.U. wrote the original draft. S.W. and H.H. reviewed and edited the article. H.H. supervised the study. K.U., A.S., and H.H. acquired funding.

## Declaration of interests

The authors declare no competing interests.

## STAR★Methods

### Key resources table

**Table:** 

REAGENT or RESOURCE	SOURCE	IDENTIFIER
**Antibodies**		

Anti-Digoxigenin-AP Fab Fragments	Roche	Cat# 11093274910; RRID: AB_514497; Lot: 11376622
Rabbit anti-γ-H2A.X (phospho S139) antibody-C-terminal	Abcam	Cat# ab228655; RRID: N/A; Lot: 1122707-1
Goat anti-Rabbit IgG (H+L) Cross-Adsorbed Secondary Antibody, Alexa Fluor™ 555	Invitrogen	Cat# A-21428; RRID: AB_2535849; Lot: 1122707-1

**Chemicals, peptides, and recombinant proteins**		

TRI-reagent LS	Molecular Research Center	Cat# TS120
Ethanol	Nacalai Tesque	Cat# 08948-25
Nuclease-free water	Nacalai Tesque	Cat# 06442-95
SuperScript IV reverse transcriptase	Thermo Fisher Scientific	Cat# 18090050
Tris(hydroxymethyl)aminomethane	Nacalai Tesque	Cat# 35434-05
KCl	Wako	Cat# 163-03545
Tween-20	Wako	Cat# 166-21115
NP-40	Roche	Cat# 11754599001
Proteinase K	Nacalai Tesque	Cat# 29442-85
Ribonuclease H	Enzymatics	Cat# Y9220L
DNA polymerase I	Enzymatics	Cat# P7050L
RNase T1	Thermo Fisher Scientific	Cat# EN0541
WGS Fragmentation Mix, 5x	Enzymatics	Cat# Y9410L
WGS Ligase, 5x	Enzymatics	Cat# L6030-W-F
KAPA HiFi HotStart ReadyMix	KAPA BIOSYSTEMS	Cat# KK2602
Paraformaldehyde	Wako	Cat# 162-16065
NaCl	Nacalai Tesque	Cat# 31320-05
Na_2_HPO_4_·12H_2_O	Wako	Cat# 196-02835
KH_2_PO_4_	Nacalai Tesque	Cat# 28721-55
Methanol	Nacalai Tesque	Cat# 21915-35
Formamide	Sigma-Aldrich	Cat# F9037
Trisodium Citrate Dihydrate	Wako	Cat# 191-01785
tRNA	Roche	Cat# 10109525001
Heparin	Sigma-Aldrich	Cat# H3393
Heat-inactivated goat serum	Invitrogen	Cat# 16210-064
Bovine serum albumin	Sigma-Aldrich	Cat# A9647
NBT/BCIP	Roche	Cat# 11681451001
MgCl_2_·6H_2_O	Nacalai Tesque	Cat# 20908-65
Levamisole	Nacalai Tesque	Cat# 20442-71
EDTA	Wako	Cat# 345-01865
Glutaraldehyde	Wako	Cat# 079-00533
Sucrose	Nacalai Tesque	Cat# 30404-45
DIG RNA Labeling Mix	Roche	Cat# 11277073910
T7 RNA polymerase	Thermo Fisher Scientific	Cat# EP0111
Triton X-100	Wako	Cat# A16046
DMSO	Nacalai Tesque	Cat# 13407-45
Acetone	Wako	Cat# 012-00343
Oil red O powder	Wako	Cat# 154-02072
Isopropanol	Nacalai Tesque	Cat# 29112-05

**Critical commercial assays**		

MEGAshortscript T7 Transcription Kit	Thermo Fisher Scientific	Cat# AM1354
mMESSAGEmMACHINE SP6 Transcription Kit	Thermo Fisher Scientific	Cat# AM1340
KAPA Taq Extra PCR Kit	KAPA BIOSYSTEMS	Cat# KK3009
KAPA SYBR FAST qPCR Kit	KAPA BIOSYSTEMS	Cat# KK4602
QuantiFluor RNA System Kit	Promega	Cat# E3310
Agilent High Sensitivity DNA kit	Agilent Technologies	Cat# 5067-4626
QuantiFluor ONE dsDNA System kit	Promega	Cat# E4870
Click-iTTM EdU Cell Proliferation Kit for Imaging, Alexa FluorTM 594 dye	Invitrogen	Cat# C10339
ApopTag Peroxidase *In Situ* Apoptosis Detection Kit	Millipore	Cat# S7101
Alexa Fluor 555 Tyramide SuperBoost Kit	Invitrogen	Cat# B40923
Glycogen Colorimetric/Fluorometric Assay Kit	BioVision	Cat# K646-100

**Deposited data**		

RNA-sequencing and quantification data	This paper	GEO: GSE276188

**Experimental models: Organisms/strains**		

Zebrafish CRISPR/Cas9 *wrn* (agu26)	This paper	N/A
Zebrafish *casper*	White et al.^49^	ZFIN: ZDB-GENO-080326-11
Zebrafish Tg(*ptf1a:EGFP*)^jh1^	Godinho et al.^50^	ZFIN: ZDB-TGCONSTRCT-070531-1

**Oligonucleotides**		

*wrn* CRISPR forward oligonucleotide, TAGGAGTGTGTGGTGGCCACAG	This paper	N/A
*wrn* CRISPR reverse oligonucleotide, AAACCTGTGGCCACCACACACT	This paper	N/A
*wrn* genotyping forward primer, TGTGTGGTGGCCA	This paper	N/A
*wrn* genotyping reverse primer, GCCCATCCCAAACGCCAC	This paper	N/A
*wrn* qPCR forward primer, TGGATTGAGCCACTGGACAA	This paper	N/A
*wrn* qPCR reverse primer, GCAGGCTGACTCACAGTCTT	This paper	N/A
*actb2* qPCR forward primer, GCCAACAGGGAAAAGATGACAC	This paper	N/A
*actb2* qPCR reverse primer, GTACGACCGGAGGCATACAG	This paper	N/A
Telomere qPCR forward primer (Tel1), GGTTTTTGAGGGTGAGGGTGAGGG TGAGGGTGAGGGT	Evans et al.^51^	N/A
Telomere qPCR reverse primer (Tel2), TCCCGACTATCCCTATCCCTATCCC TATCCCTATCCCTA	Evans et al.^51^	N/A
*c-fos* qPCR forward primer, CAGCTCCACCACAGTGAAGA	Evans et al.^51^	N/A
*c-fos* qPCR reverse primer, GCTCCAGGTCAGTGTTAGCC	Evans et al.^51^	N/A
*wrn in situ* hybridization forward primer, AGAGCACAAGAGCTTGAGGC	This paper	N/A
*wrn in situ* hybridization reverse primer, AAGCCAGCTCCATTCCACTG	This paper	N/A

**Software and algorithms**		

ImageJ (version 1.54)	NIH	https://imagej.nih.gov/ij
fastp (version 0.21.0)	Chen et al.^52^	https://github.com/OpenGene/fastp
BWA-MEN (version 0.7.17-r1188)	Li and Durbin.^53^	https://github.com/j-levy/bwa
Salmon (version v0.12.0)	Patro et al.^54^	https://combine-lab.github.io/salmon/
R statistical software (version 4.4.1)	R Core Team	https://www.r-project.org/
Seurat (version 5.1.0)	Hao et al.^55^	https://satijalab.org/seurat/
clusterProfiler (version 4.12.0)	Wu et al.^56^	https://github.com/YuLab-SMU/clusterProfiler

**Other**		

Gemma Micro ZF 75	Funakoshi	Cat# 10818935
Otohime B2	Marubeni Nissin Feed	N/A
Gemma Micro ZF 300	Funakoshi	Cat# 10818955
AcroPrep Advance 96-well Long Tip Filter Plate for Nucleic Acid Binding	Pall	Cat# 8133
BioMasher II	Nippi	Cat# 320102
Ampure XP beads	Beckman Coulter	Cat# A63880
O.C.T compound	Sakura Finetec Japan	Cat# 4583

### Experimental model and study participant details

Zebrafish (*Danio rerio*) were used as the experimental animal model in this study. Wild-type (*wrn*^+/+^), heterozygous (*wrn*^+/-^), and homozygous mutant (*wrn*^-/-^; *wrn*^agu26^) zebrafish were maintained on a casper background (*nacre*^-/-^, *roy*^-/-^) to facilitate *in vivo* visualization.^49^ The following previously reported transgenic line was used: Tg(*ptf1a:EGFP*)^jh1^.^50^ Zebrafish were reared and maintained in 1.7 L tanks in a recirculating Meito System (Meito System) under a 14 h light and 10 h dark photoperiod, according to standard protocols. Zebrafish larvae were raised in 90 mm dish until 5 dpf and then placed in the recirculation system at 5 dpf. Larvae fish were fed Gemma Micro ZF 75 (Funakoshi) and paramecia twice a day from 5 dpf to 1 month post-fertilization (mpf). Juvenile fish (1-3 mpf) were fed Gemma Micro ZF 75 and brine shrimp (Tokai Guppy) twice a day. Adult fish (3 mpf-) were fed Otohime B2 (Marubeni Nissin Feed) or Gemma Micro ZF 300 (Funakoshi) and brine shrimp twice a day.

Experiments were performed at defined developmental stages, including larval (5-30 dpf), juvenile (30-90 dpf), and adult stages as indicated in each experiment. Sex could not be determined at larval stages and therefore sex-specific analyses were not performed at these time points. For juvenile and adult fish, males and females were used without distinction.

This study was approved by the Animal Care and Ethics Committee of Aoyama Gakuin University (A17-25) and carried out according to the Animal Research Reporting of In VIVO Experiments (ARRIVE) guidelines and relevant regulations. No human participants, cell lines, or primary cell cultures were used in this study.

### Method details

#### Generation of *wrn* mutant zebrafish and genotyping

The CRISPR/Cas9 target was selected in exon 19 of the zebrafish *wrn* gene. To generate a gRNA construct, *wrn* CRISPR forward and reverse oligonucleotides (TAGGAGTGTGTGGTGGCCACAG and AAACCTGTGGCCACCACACACT, respectively) were annealed and subcloned into the BsaI restriction site of pDR274. The gRNAs were synthesized from HindIII-digested pDR274-zWRN plasmids using the MEGAshortscript T7 Transcription Kit (Thermo Fisher Scientific). The Cas9 RNA was synthesized from pCS2+hSpCas9, which contains human codon–optimized *S. pyogenes* Cas9 cDNA, using the mMESSAGE mMACHINE SP6 Transcription Kit (Thermo Fisher Scientific). A solution containing 20 ng/μl gRNA and 200 ng/μl Cas9 RNA was injected into zebrafish embryos at the one-cell stage. To detect insertion/deletion mutations, the target regions were amplified using PCR and subjected to heteroduplex mobility analysis. The 2-bp deletion (*wrn*^agu26^) was confirmed by sequencing after subcloning the target region. For genotyping, the missense region of *wrn* gene was amplified by genomic PCR using the KAPA Taq Extra PCR Kit (KAPA BIOSYSTEMS) and forward and reverse primers (TGTGTGGTGGCCA and GCCCATCCCAAACGCCAC, respectively) in ProFlex PCR Systems (Thermo Fisher Scientific). The following program was used for amplification: 94 °C 2 min; 98 °C 10 s, 60 °C 20 s, 72 °C 30 s, 35 cycles; 72 °C 1 min; 4 °C forever. The PCR products were separated by a 15% polyacrylamide gel electrophoresis at 300 V for 55 min. Gel images were captured using Printgraph AE-6933FXCF (Atto). The *wrn*^agu26^ line was maintained as heterozygotes by outcrossing to wild-type AB zebrafish. Homozygous mutants obtained from crosses of heterozygotes from different generations consistently exhibit the same phenotypic spectrum and severity.

#### Imaging

Images were captured using a microscope digital camera DP74 (Olympus). Measurements of body length and pancreatic exocrine surface area were performed using ImageJ (version 1.54).

#### Quantitative PCR

For *wrn* qPCR, RNA extraction from 5 dpf zebrafish larvae and RNA purification were performed as previously described.^38^ Briefly, RNA extraction from 5 dpf larvae was conducted by vortexing the sample with TRI-reagent LS (Molecular Research Center) in an 8-strip tube for 1 min at room temperature (RT). Equal amounts of 99.5% ethanol were added to the RNA lysates and mixed together. The mixtures were applied to AcroPrep Advance 96-well Long Tip Filter Plate for Nucleic Acid Binding (Pall) and centrifuged at 1,300 x g for 4 min at RT. The RNA adsorbed by the filter was washed twice with 99.5% ethanol and once with 80% ethanol and finally eluted with nuclease-free water. Reverse transcription was performed using SuperScript IV reverse transcriptase (Thermo Fisher Scientific) and oligo(dT) primers. Quantitative PCR of *wrn* was carried out using the KAPA SYBR FAST qPCR Kit (KAPA BIOSYSTEMS) and forward and reverse primers (*wrn* forward: TGGATTGAGCCACTGGACAA; reverse: GCAGGCTGACTCACAGTCTT and *actb2* forward: GCCAACAGGGAAAAGATGACAC; reverse: GTACGACCGGAGGCATACAG, respectively) in QuantStudio 5 Real-Time PCR Systems (Thermo Fisher Scientific). The following program was used for amplification: 95 °C 20 sec; 95 °C 1 s, 55 °C 20 sec, 45 cycles.

For telomere qPCR, genome DNA was extracted from 7 dpf zebrafish larvae with Lysis Buffer (10 mM Tris-HCl [pH 8.0], 10 mM KCl, 0.3% Tween-20, 0.3% NP-40) and 0.5 mg/ml Proteinase K for at 56 °C for 3 h. Genome DNA was extracted and purified by sequential extraction with phenol/chloroform/isoamyl alcohol and chloroform/isoamyl alcohol, followed by ethanol precipitation. The primer and reaction protocol were used described method.^51^ Quantitative PCR of telomere was carried out using the KAPA SYBR FAST qPCR Kit and forward and reverse primers (telomere forward: GGTTTTTGAGGGTGAGGGTGAGGGTGAGGGTGAGGGT; reverse: TCCCGACTATCCCTATCCCTATCCCTATCCCTATCCCTA and *c-fos* forward: CAGCTCCACCACAGTGAAGA; reverse: GCTCCAGGTCAGTGTTAGCC, respectively) in QuantStudio 5 Systems. The following program was used for amplification: 95 °C 10 min; 95 °C 15 s, 54 °C 2 min, 40 cycles.

Relative expression levels of *wrn* were calculated using the ΔCt method, with *actb2* serving as the internal control. Briefly, Ct values obtained from each sample were normalized to *actb2*, and the relative expression was calculated as 2^ΔCt^ compared to the *wrn*^+/+^. For telomere qPCR, the relative telomere length was determined as the ratio of telomere repeat copy number to the single-copy reference gene (*c-fos*), following the method described previously.^51^ The relative telomere length was calculated as 1/(Ct_telomere/Ct_*c-fos*).

#### Survival test

Larvae zebrafish that were obtained by a cross of *wrn*^+/-^ fish were reared under regular care. Dead larvae/juveniles were collected every day from 5 to 88 dpf and stored at -30 °C until genotyping. Dead individuals and surviving fish were genotyped at 88 dpf. In the survival assay under starvation conditions, dead individuals were subjected to genotyping at 14 dpf as the endpoint of the assay. In the survival assay under extra feeding conditions, larvae were reared in 100 mm cell culture dishes and fed three times the normal amount of paramecia and Gemma Micro ZF 75 twice daily. Dead and surviving individuals were genotyped at 21 dpf as the endpoint of the assay.

#### RNA extraction for transcriptome analysis

RNA extraction from larvae and juvenile zebrafish and RNA purification method were see above. RNA extraction from to 4-12 dpf larvae was conducted by vortexing the sample with TRI-reagent LS in an 8-strip tube for 1 min at RT. RNA extraction from to 15-35 dpf larvae/juveniles was conducted by homogenizing samples in TRI-reagent LS using BioMasher II (Nippi) for 1 min at RT. RNA concentration was determined using Quantus Fluorometer (Promega) with the QuantiFluor RNA System Kit (Promega) according to the manufacturer’s instructions. RNA was diluted to a final concentration of 2 ng/μl and used to prepare library for transcriptome analysis.

#### Library preparation

Library preparation was conducted using Lasy-Seq version 1.1 method (https://sites.google.com/view/lasy-seq/). Briefly, reverse transcription was performed using SuperScript IV reverse transcriptase and indexed reverse transcription primers. The following program was used for reverse transcription: 65 °C 10 min; 80 °C 15 min; 4 °C forever in ProFlex PCR Systems. Reverse transcription products were pooled and purified using an equal volume of AMpure XP beads (Beckman Coulter), according to the manufacturer’s instruction. Second-strand synthesis was conducted using Ribonuclease H (Enzymatics) and DNA polymerase I (Enzymatics) at 16 °C for 2 h. Synthetic double-strand DNA (dsDNA) were treated with RNase T1 (Thermo Fisher Scientific) at 37 °C for 5 min. Fragmentation was conducted using 5x WGS Fragmentation Mix (Enzymatics) at 32 °C for 6.5 min followed by heat inactivation at 65 °C for 30 min. Ligation was conducted using 5x WGS Ligase (Enzymatics) at 20 °C for 15 min. Library amplification was conducted using KAPA HiFi HotStart ReadyMix (KAPA BIOSYSTEMS) in QuantStudio 5 Real-Time PCR Systems. The following program was used for amplification: 95 °C 5 min; 98 °C 20 s, 60 °C 15 s, 72 °C 40 s, 12 cycles; 72 °C 3 min; 4 °C forever. The library was subjected to electrophoresis using Bioanalyzer 2100 (Agilent Technologies) with the Agilent High Sensitivity DNA kit (Agilent Technologies) according to the manufacturer’s instructions. The library concentration was determined using Quantus Fluorometer with the QuantiFluor ONE dsDNA System kit (Promega) and diluted to 4 ng/μl. Sequencing of 150-bp paired-end reads was conducted using HiSeq X Ten (Illumina).

#### Mapping, gene quantification and analysis

Mapping and gene quantification were performed as previously described.^57^ Briefly, only read 1 reads were processed with fastp (version 0.21.0).^52^ The trimmed reads were then mapped to the zebrafish reference sequences of Danio_rerio.GRCz11.101 using BWA-MEM (version 0.7.17-r1188)^53^ with the default parameters. The read count for each gene was calculated with Salmon using -l IU, which specifies the library type (version v0.12.0).^54^ Thereafter, the sum of the read counts per gene was calculated using R (version 4.4.1) (https://www.r-project.org/). Read counts were normalized using the “NormalizeData” function with the default parameters in Seurat (version 5.1.0).^55^ The four samples with the lowest number of RNA reads were excluded using “Subset” with nCount_RNA < 10^5.5^. For principal component analysis (PCA), the normalized read counts were centered but not scaled using the “ScaleData” function with the default parameters except for do.scale = F. Then, PCA was performed using the “RunPCA” function for genes with high dispersion, which were selected using the “FindVariableFeatures” function with the default parameters. Clustering was performed using the “FindClusters” with resolution = 3. Gene Ontology enrichment analysis was performed using the “enrichGO” function implemented in the clusterProfiler (version 4.12.0).^56^ Statistical significance was determined using the Benjamini-Hochberg method for multiple testing correction, with a q-value threshold of < 0.05. Organ-specific single-cell transcriptome data were obtained from the Zebrafish Cell Landscape.^58^ Sequencing and quantification data were deposited in the Gene Expression Omnibus (GEO, accession number: GSE276188).

#### Histological analysis

Zebrafish larvae were fixed in 4% PFA in PBS (pH 7.4; 1.37 M NaCl, 81 mM Na_2_HPO_4_·12H_2_O, 27 mM KCl, 15 mM KH_2_PO_4_) overnight at 4 °C and then embedded in paraffin. Paraffin-embedded tissues were sectioned in an axial orientation at a thickness of 4 μm. Hematoxylin and eosin staining was performed on deparaffinized tissue sections, as previously described.^59^ Measurement of intestinal villi length and intestine size were used ImageJ (version 1.54).

#### *In situ* hybridization

Zebrafish larvae Tg(*ptf1a*:*EGFP*) were fixed in 4% PFA in PBS overnight at 4 °C, washed with PBST (1x PBS, 0.1% Tween-20), dehydrated through a methanol series, and stored in 100% methanol at -30 °C. For *in situ* hybridization, samples were gradually rehydrated and treated with 10 μg/ml Proteinase K for 2 h at RT, followed by postfixation in 4% PFA in PBS for 20 min at 4 °C. Prehybridization was performed in Hybridization Buffer (50% formamide, 5x SSC [pH 4.5; 3 M NaCl, 300 mM Trisodium Citrate Dihydrate], 0.1% Tween-20, 500 μg/ml tRNA, 50 μg/ml heparin) at 68 °C for 30 min. Digoxigenin (DIG)-labeled RNA probes were then applied, and hybridization was carried out overnight at 68 °C. After hybridization, samples were washed sequentially with 2x SSCT (2x SSC, 0.1% Tween-20), 0.2x SSCT (0.2x SSC, 0.1% Tween-20), and PBST, and then blocked with Blocking Buffer (1x PBS, 0.1% Tween-20, 2% heat-inactivated goat serum, 2% bovine serum albumin) at RT. Samples were incubated with anti-DIG-AP Fab fragments (1:2000, Roche) overnight at 4 °C. Colorimetric detection was performed using NBT/BCIP substrate (1:100, Roche) in NTMT buffer (100 mM NaCl, 100 mM Tris-HCl [pH 9.5], 50 mM MgCl_2_·6H_2_O, 0.1% Tween-20, 1 mM levamisole) at RT in the dark. The staining reaction was terminated by washing with TET buffer (10 mM Tris-HCl [pH 8.0], 5 mM EDTA [pH 8.0], 0.1% Tween-20) followed by PBST. Samples were then post-fixed in 4% PFA containing 0.25% glutaraldehyde in PBS overnight at 4 °C. Subsequently, the samples were cryoprotected in 30% sucrose in PBS overnight at 4 °C, embedded in O.C.T. compound (Sakura Finetec Japan), and sectioned axially at a thickness of 16 μm. Images were captured using a microscope digital camera DP74 (Olympus). The RNA probe targeting the *wrn* transcript was synthesized using a PCR-amplified template with the following forward and reverse primers (AGAGCACAAGAGCTTGAGGC and AAGCCAGCTCCATTCCACTG, respectively). The amplified fragment was purified and used as a template for *in vitro* transcription with DIG RNA Labeling Mix (Roche) and T7 RNA polymerase (Thermo Fisher Scientific).

#### Immunostaining

Zebrafish larvae Tg(*ptf1a:EGFP*); *casper* were fixed in 4% PFA in PBS overnight at 4 °C, followed by dehydration in 30% sucrose in PBS overnight at 4 °C. Samples were embedded in O.C.T. compound (SAKURA) and sectioned axially at a thickness of 16 μm. Sections were washed three times with PBST for 5 min at RT. Blocking was performed using Blocking Buffer (1x PBS, 5% heat-inactivated goat serum, 1% bovine serum albumin, 0.5% Triton X-100, 1% DMSO) for 1 hour at RT. Sections were then incubated with primary antibody solution overnight at 4 °C. After washing five times with PBST for 5 min at RT, sections were incubated with secondary antibody solution overnight at 4 °C in the dark. The following day, sections were washed five times with PBST for 5 min at RT, protected from light. Images were captured using a confocal microscope Stellaris 5 (Leica). The primary antibody used was rabbit anti-γ-H2A.X (phospho S139) antibody-C-terminal (1:200, Abcam). The secondary antibody used was Goat anti-Rabbit IgG (H+L) Cross-Adsorbed Secondary Antibody, Alexa Fluor™ 555 (1:1000, Invitrogen). Quantification of staining signals were used ImageJ (version 1.54).

#### EdU staining

Zebrafish larvae Tg(*ptf1a:EGFP*); *casper* were reared for one day in EdU water (0.5 mM EdU, 0.5% DMSO). EdU-treated larvae were fixed in 4% PFA in PBS overnight at 4 °C, followed by dehydration in 30% sucrose in PBS overnight at 4 °C. Samples were embedded in O.C.T compound and sectioned axially at a thickness of 16 μm. To detect EdU cells, the Click-iT^TM^ EdU Cell Proliferation Kit for Imaging, Alexa Fluor^TM^ 594 dye (Invitrogen) was used according to the manufacturer’s instruction. Images were captured using a confocal microscope Stellaris 5. Quantification of staining signals were used ImageJ (version 1.54).

#### TUNEL staining

Zebrafish larvae Tg(*ptf1a:EGFP*); *casper* were fixed in 4% PFA in PBS overnight at 4 °C, followed by dehydration in 30% sucrose in PBS overnight at 4 °C. Samples were embedded in O.C.T. compound and sectioned axially at a thickness of 16 μm. To ensure delipidation, the sections were treated with a series of PBST, Ethanol and Acetone mixtures. The sections were treated with 25 μg/ml Proteinase K for 30 min at RT and washed three times with PBST for 3 min at RT. To detect apoptotic cell, the ApopTag Peroxidase *In Situ* Apoptosis Detection Kit (Millipore) and the Alexa Fluor™ 555 Tyramide SuperBoost™ Kit (Invitrogen) were used according to the manufacturer’s manual. Images were captured using a confocal microscope Stellaris 5. Quantification of staining signals were used ImageJ (version 1.54).

#### Lipid staining

Zebrafish larvae *casper* were fixed in 4% PFA in PBS overnight at 4 °C. The fixed samples were washed three times with PBST for 5 min at RT. Next, a 0.5% Oil Red O solution (Oil Red O powder, 60% isopropanol) was added to the samples and incubated for 30 min at RT. The staining samples were washed three times with PBST for 5 min at RT and washed once with 60% isopropanol for 5 min at RT. Samples were post-fixed in 4% PFA in PBS for 10 min at RT. Images were captured using a microscope digital camera DP74. Quantification of staining signal intensity was used ImageJ (version 1.54).

#### Measurement of glucose and glycogen levels

Zebrafish larvae Tg(*ptf1a:EGFP*)*; casper* were homogenized using the manual homogenizer BioMasher II (Nippi). Distilled water was added, and the samples were boiled at 85 °C for 10 min to inactivate catabolic enzymes of glucose and glycogen. Boiled samples were subjected to glucose and glycogen measurement using the Glycogen Colorimetric/Fluorometric Assay Kit (BioVision) according to the manufacturer’s instructions. Fluorescent detection was performed using a laboratory-customized Fluorescence/Raman microscope (Nikon, TE2000) equipped with a cw 532 nm diode-pumped solid-state laser (Spectra-Physics, Millennia V), a motorized microplate stage, and a spectrometer (Bunkoukeiki, MK-300 and Princeton Instruments, Pixis 100B).

### Quantification and statistical analysis

All sample sizes were included in the figure legends. Quantitative data are presented as mean ± standard deviation (SD). All mean ± SD were shown in the supplymental tables. All error bars in the figures represent SD. A Kaplan-Meier log-rank test was used to compare survival rates between two groups (Figures 1B and 5G). A two-tailed Student’s t test was used for comparisons between two groups (Figures 1G, 3O, 4F, 4G, 4M, 4N, and 5N). A Welch’s t test was used for comparisons between two groups with unequal variances (Figures 2J–2M, S3F–S3H, and S5A–S5L). A Wilcoxon rank-sum test was applied for non-parametric comparisons between two groups (Figure 4U). For comparisons among three groups, Welch's ANOVA followed by the Games-Howell post hoc test was performed (Figures 5A, 5B, and 5F). One-way ANOVA followed by Tukey’s Honestly Significant Difference (HSD) test was used to compare the three groups across multiple time points (Figures 3O–3Q, 4F, 4G, 4M, 4N, 5N, S1D, and S4). A Kruskal-Wallis test followed by Dunn’s multiple comparisons test with Holm correction was applied for non-parametric comparisons among three groups (Figures 4T and 4U). ∗*P*<0.05, ∗∗*P*<0.01, ∗∗∗*P*<0.001, ns : not significant. *P*-value in gene expression change (Figures 2J–2M, S3F–S3H, and S5A–S5L) were shown in Table S5.
