# Supplementary material for: Deficiency of Werner RecQ-type DNA helicase causes premature malnutrition in zebrafish
Source: iScience. 2026 Jan 21;29(3):114760. doi: 10.1016/j.isci.2026.114760 (PMC12924724; doi:10.1016/j.isci.2026.114760)
Supplement: Document S1. Figures S1–S5 and Tables S1–S11 [file mmc1.pdf]

iScience, Volume *29*

Supplemental information

Deficiency of Werner RecQ-type DNA helicase causes premature malnutrition in zebrafish

Kota Ujibe, Makoto Kashima, Miku Kataoka, Rintaro Shimada, Masashige Okamoto, Isao Kobayashi, Seiji Wada, Hiroki Matsuda, Akira Sakamoto, and Hiromi Hirata

Supplemental information

Deficiency of Werner RecQ-type DNA helicase causes premature malnutrition in zebrafish

Kota Ujibe, Makoto Kashima, Miku Kataoka, Rintaro Shimada, Masashige Okamoto, Isao Kobayashi, Seiji Wada, Hiroki Matsuda, Akira Sakamoto, and Hiromi Hirata

Ujibe et al. Figure S1

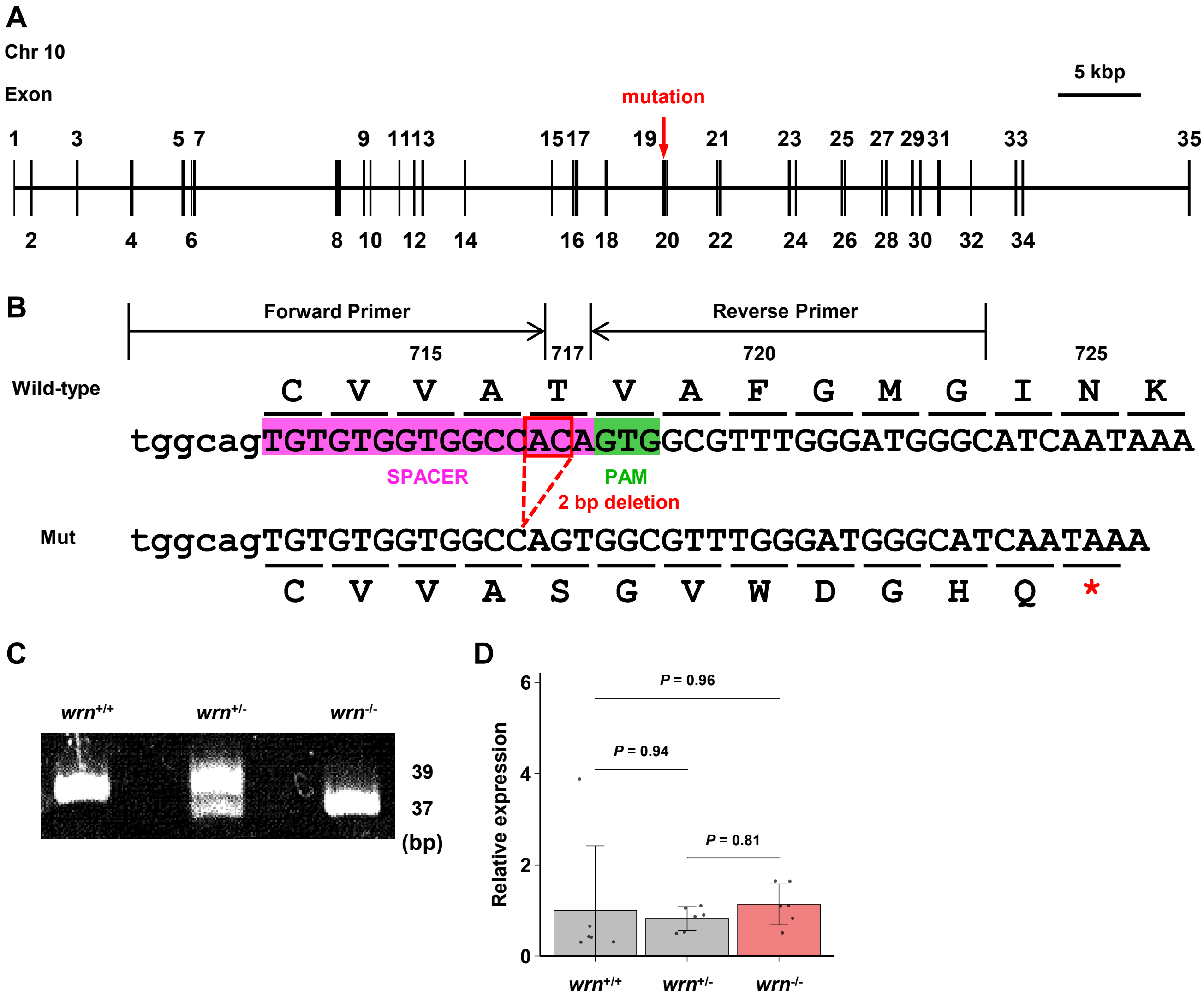

Figure S1. Generation of *wrn* mutant zebrafish.
(A) Exon-intron structure of the zebrafish *wrn* gene. The mutation site is located in exon 19. Scale bar, 5 kb. (B) CRISPR/Cas9-mediated 2-bp deletion in *wrn* gene. This deletion induces a frameshift after the alanine residue at position 716 in the helicase domain, resulting in the addition of 8 aberrant amino acids, followed by a stop codon (T717Sfs*9). (C) Genotyping of *wrn*$^{+/+}$, *wrn*$^{+/-}$, and *wrn*$^{-/-}$ by polyacrylamide gel electrophoresis. The PCR products for the wild-type and deletion alleles were 39 bp and 37 bp, respectively. (D) Relative expression of *wrn* in *wrn*$^{+/+}$ (n = 6), *wrn*$^{+/-}$ (n = 6), and *wrn*$^{-/-}$ (n = 6) larvae at 5 dpf. Expression levels were normalized to *actb2* (β-actin) as an internal control. Statistical significance was assessed using one-way ANOVA followed by Tukey's Honestly Significant Difference (HSD) test. Data are presented as mean ± standard deviation (SD). The detailed mean ± SD are shown in Table S1.

Ujibe et al. Figure S2

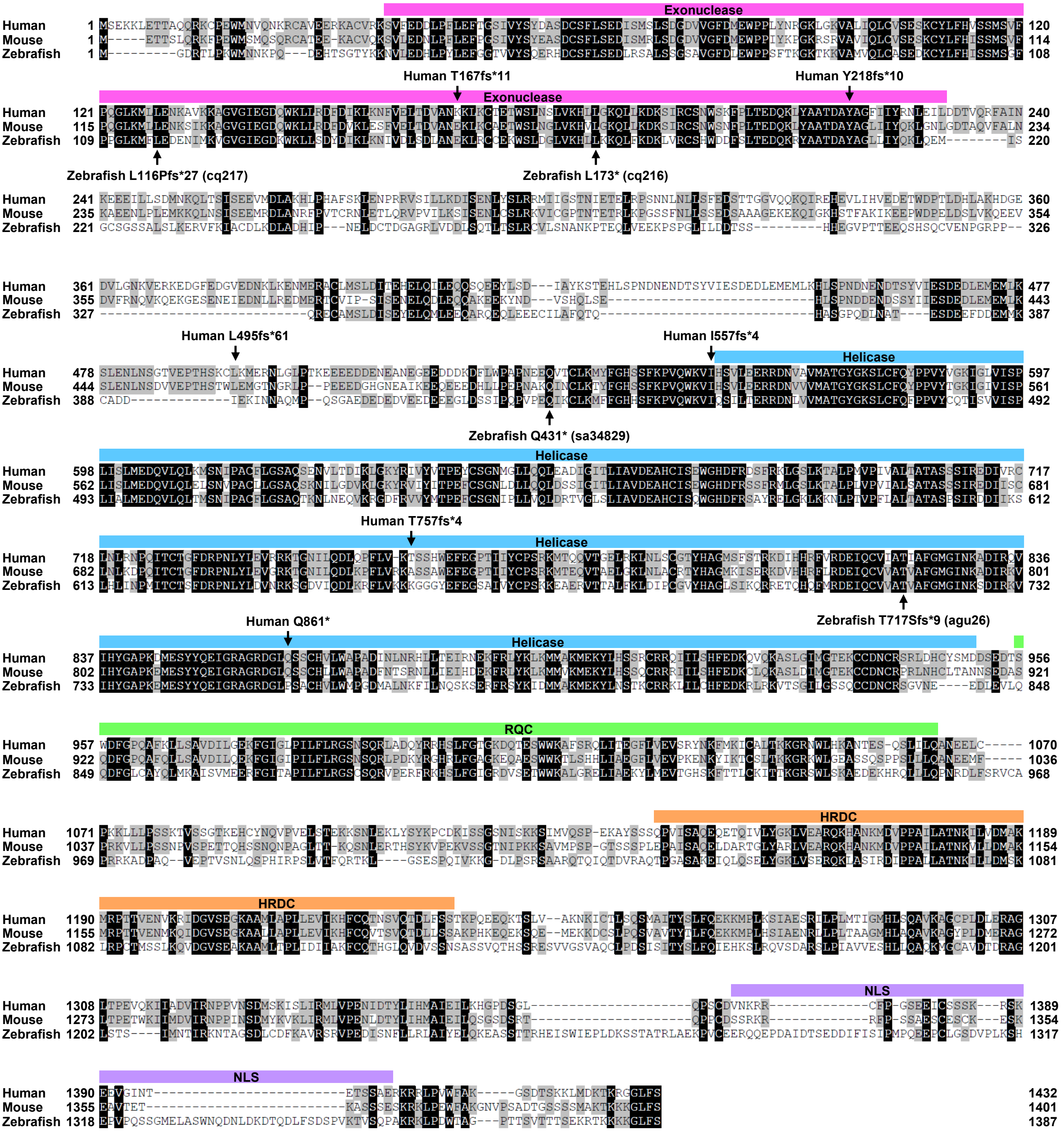

Figure S2. Amino acid alignment of WRN proteins in vertebrate.
Alignment of human (NP_000544.2), mouse (NP_035851.3), and zebrafish (XP_073770491.1) WRN proteins. The zebrafish mutants generated in this study carries a frameshift in the helicase domain (agu26: T717S fs*9). Previously reported zebrafish mutants harbor nonsense mutations in the exonuclease domain (cq217: L116Pfs*27; cq216: L173*) or upstream of the helicase domain (sa34829: Q431*). Mutations identified in human Werner syndrome patients that are located near the corresponding regions of each zebrafish mutant are also shown (Huang et al., 2006).

Ujibe et al. Figure S3

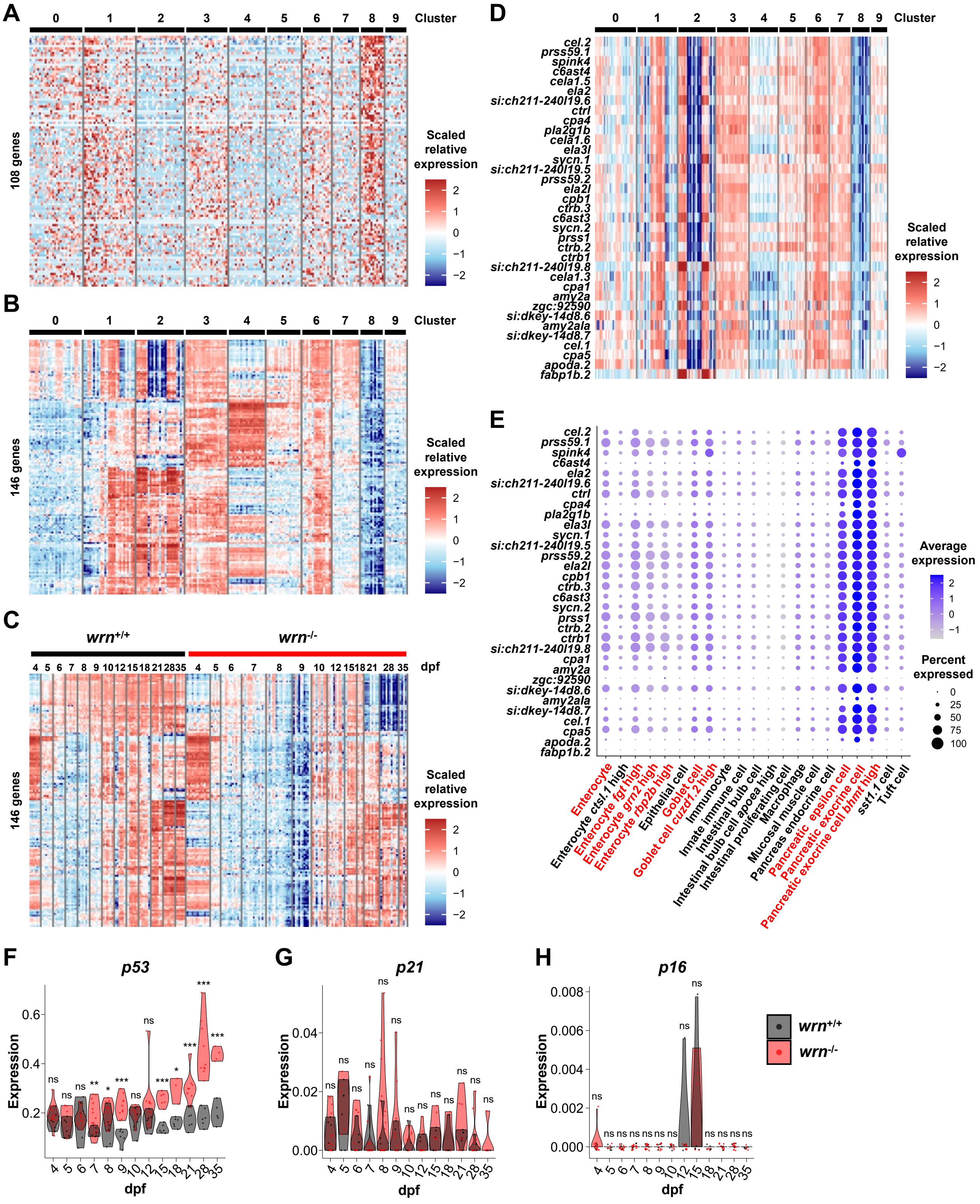

Figure S3. Transcriptome identifies downregulation of pancreatic exocrine and intestine genes and upregulation of DNA damage marker in *wrn*$^{-/-}$ larvae.
(A) Heatmap of 108 genes that were upregulated in Cluster 8. (B) Heatmap of 146 genes that were downregulated in prematurely dying *wrn*$^{-/-}$, organized by sample clusters. (C) Heatmap of 146 genes, organized by developmental stage. (D) Heatmap of 35 genes downregulated in *wrn*$^{-/-}$ of Cluster 2 and 8. (E) Cell type-specific expression profiles of 32 genes (excluding *cela1.3*, *cela1.5*, and *cela1.6*) in pancreas and intestine based on the Zebrafish Cell Landscape. (F-H) Expression of DNA damage and cell senescence marker in *wrn*$^{+/+}$ (black) and *wrn*$^{-/-}$ (red) zebrafish. DNA damage marker: *p53* (F); cell senescence marker: *p21* (G) and *p16* (H). Statistical significance was assessed using Welch's t-test for each dpf. **P*<0.05, ***P*<0.01, ****P*<0.001, ns : not significant. The detailed *P*-values are shown in Table 5.

Ujibe et al. Figure S4

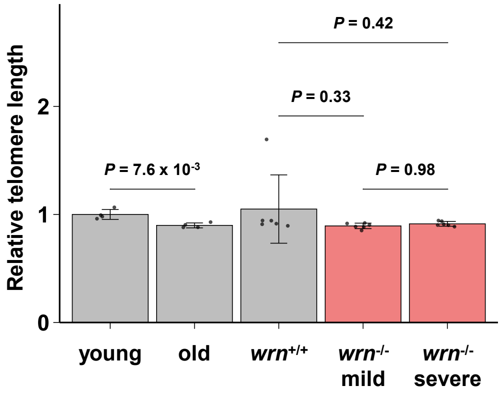

Figure S4. Telomere length in *wrn*$^{-/-}$_severe were not shorter than *wrn*$^{+/+}$ and *wrn*$^{-/-}$_mild at 7 dpf. Relative telomere length of young (4.5 months post-fertilization (mpf), *n* = 4), old (3 to 5 years post-fertilization (ypf), *n* = 4), *wrn*$^{+/+}$ (*n* = 6), *wrn*$^{-/-}$_mild (*n* = 6), and *wrn*$^{-/-}$_severe (*n* = 6) larvae at 7 dpf. The *c-fos* gene was used as a single-copy reference gene. Relative telomere length = 1/(Ct_telomere/Ct_*c-fos*). Statistical significance between young and old was assessed using an unpaired two-tailed Student's t-test, and that of *wrn* at 7 dpf was assessed using one-way ANOVA followed by Tukey's HSD test. Data are presented as mean ± SD. The detailed mean ± SD are shown in Table S9.

Ujibe et al. Figure S5

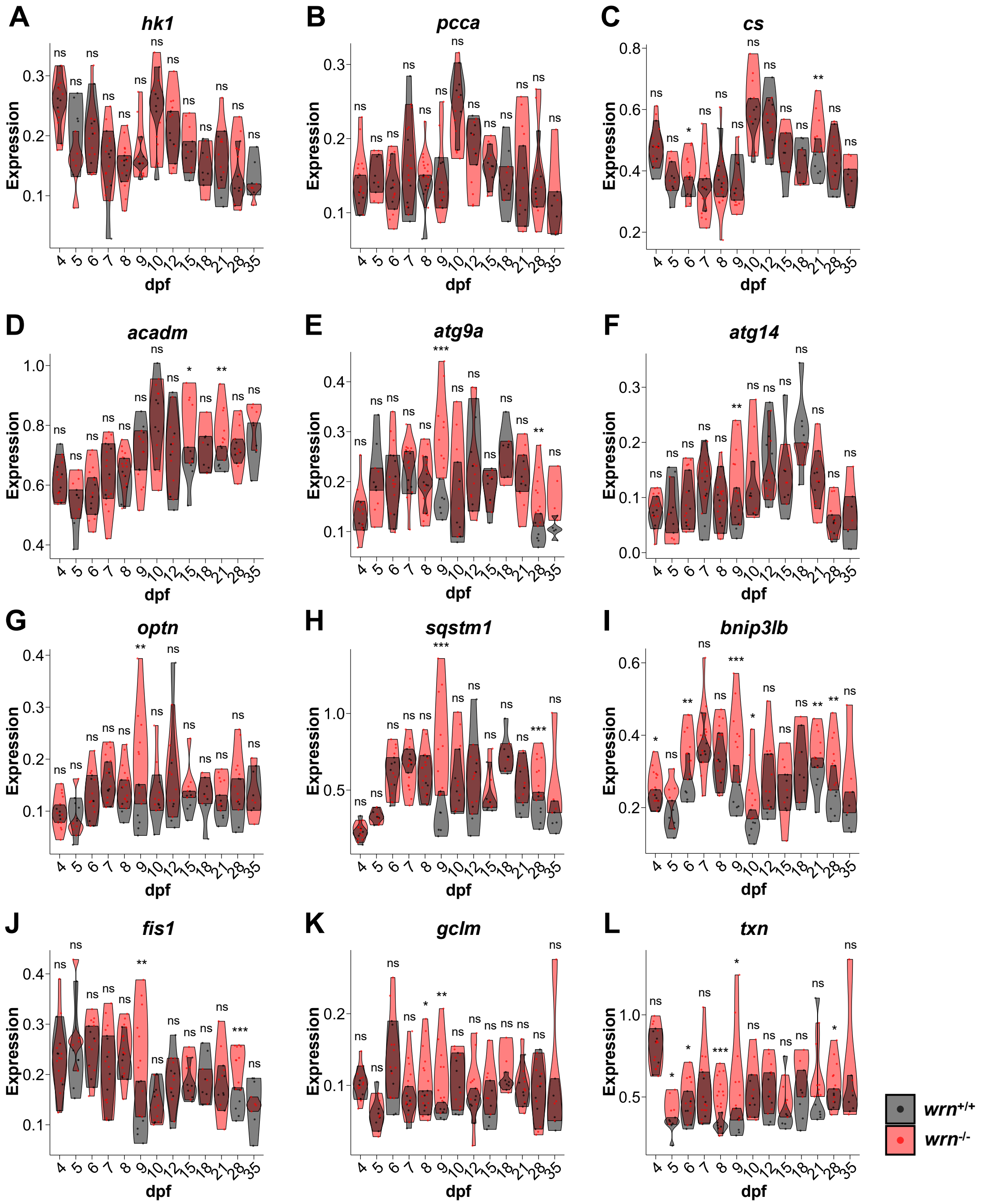

Figure S5. Expression of metabolic enzymes remains unchanged, and autophagy, mitophagy and oxidative stress markers are upregulated in prematurely dying *wrn*$^{-/-}$ larvae.

(A-D) Expression of metabolic enzymes in *wrn*$^{+/+}$ (black) and *wrn*$^{-/-}$ (red) zebrafish. (A) Glycolytic enzyme hexokinase 1 (*hk1*). (B) Gluconeogenic enzyme propionyl-CoA carboxylase a (*pcca*). (C) Tricarboxylic acid cycle enzyme citrate synthase (*cs*). (D) Lipid metabolism enzymes acyl-CoA dehydrogenase medium chain (*acadm*). (E-H) Expression of autophagy genes in *wrn*$^{+/+}$ (black) and *wrn*$^{-/-}$ (red) zebrafish. Autophagy regulator (E) autophagy related 9A (*atg9a*), (F) autophagy related 14 (*atg14*), (G) optineurin (*optn*), and (H) sequestosome 1 (*sqstm1*). (I, J) Expression of mitophagy markers in *wrn*$^{+/+}$ (black) and *wrn*$^{-/-}$ (red) zebrafish. (I) BCL2 interacting protein 3 like b (*bnip3lb*) and (J) fission 1 (*fis1*). (K, L) Expression of oxidative stress markers in *wrn*$^{+/+}$ (black) and *wrn*$^{-/-}$ (red) zebrafish. (K) glutamate-cysteine ligase modifier subunit (*gclm*) and (L) thioredoxin (*txn*). Statistical significance was assessed using Welch's t-test for each dpf. *$P<0.05$, **$P<0.01$, ***$P<0.001$, ns : not significant. The detailed *P*-values are shown in Table 5.

Table S1. Mean ± SD of expression of *wrn* in *wrn*$^{+/+}$, *wrn*$^{+/-}$, and *wrn*$^{-/-}$ larvae at 5 dpf.

	wrn$^{+/+}$	*wrn*$^{+/-}$	*wrn*$^{-/-}$
Expression of *wrn*	1.00 ± 1.42	0.82 ± 0.26	1.14 ± 0.45

Ujibe et al. Table S2

Table S2. Mean ± SD of body length of *wrn*$^{+/+}$ and *wrn*$^{-/-}$ zebrafish at each stage.

Body length (mm)	*wrn*$^{+/+}$	*wrn*$^{-/-}$_mild
21 dpf	5.82 ± 0.71	5.69 ± 0.67
75 dpf	16.97 ± 1.62	11.96 ± 1.98

Ujibe et al. Table S3

Table S3. The number of samples for time-course transcriptome analysis.

dpf	4	5	6	7	8	9	10	12	15	18	21	28	35	Total
wrn$^{+/+}$	6	6	6	6	6	6	6	6	6	6	6	6	5	77
wrn$^{-/-}$	13	5	11	13	14	10	8	9	6	3	8	10	4	114

Ujibe et al. Table S4

Table S4. List of 35 genes downregulated in *wrn*$^{-/-}$ zebrafish.

	Gene name	
1	*cel.2*	Carboxyl ester lipase, tandem duplicate 2
2	*prss59.1*	Serine protease 59, tandem duplicate 1
3	*spink4*	Serine peptidase inhibitor, Kazal type 4
4	*c6ast4*	Six-cysteine containing astacin protease 4
5	*cela1.5*	Chymotrypsin like elastase family member 1, tandem duplicate 5
6	*ela2*	Elastase 2
7	*si:ch211-240l19.6*	None
8	*ctrl*	Chymotrypsin-like
9	*cpa4*	Carboxypeptidase A4
10	*pla2g1b*	Phospholipase A2, group IB
11	*cela1.6*	Chymotrypsin like elastase family member 1, tandem duplicate 6
12	*ela3l*	Elastase 3 like
13	*sycn.1*	Syncollin, tandem duplicate 1
14	*si:ch211-240l19.5*	None
15	*prss59.2*	Serine protease 59, tandem duplicate 2
16	*ela2l*	Elastase 2 like
17	*cpb1*	Carboxypeptidase B1
18	*ctrb.3*	Chymotrypsinogen B, tandem duplicate 3
19	*c6ast3*	Six-cysteine containing astacin protease 3
20	*sycn.2*	Syncollin, tandem duplicate 2
21	*prss1*	Serine protease 1
22	*ctrb.2*	Chymotrypsinogen B, tandem duplicate 2
23	*ctrb.1*	Chymotrypsinogen B, tandem duplicate 1
24	*si:ch211-240l19.8*	None
25	*cela1.3*	Chymotrypsin like elastase family member 1, tandem duplicate 3
26	*cpa1*	Carboxypeptidase A1
27	*amy2a*	Amylase alpha 2A
28	*zgc:92590*	None
29	*si:dkey-14d8.6*	None
30	*amy2ala*	Amylase alpha 2A-like 2
31	*si:dkey-14d8.7*	None
32	*cel.1*	Carboxyl ester lipase, tandem duplicate 1
33	*cpa5*	Carboxypeptidase A5
34	*apoda.2*	Apolipoprotein Da, duplicate 2
35	*fabp1b.2*	Fatty acid binding protein 1b, liver, tandem duplicate 2

Ujibe et al. Table S5

Table S5. *P*-values of differences in transcript levels between *wrn*$^{+/+}$ and *wrn*$^{-/-}$ zebrafish at each stage.

dpf	4	5	6	7	8	9	10	12	15	18	21	28	35
prss1	0.20	0.20	3.6×10^{-2}	7.1×10^{-4}	1.9×10^{-4}	2.0×10^{-4}	4.6×10^{-2}	0.01	4.8×10^{-2}	6.5×10^{-4}	9.7×10^{-6}	3.8×10^{-7}	9.5×10^{-3}
amy2a	0.82	4.6×10^{-2}	0.01	4.8×10^{-4}	4.5×10^{-5}	2.2×10^{-5}	0.02	2.1×10^{-3}	0.02	8.6×10^{-3}	5.4×10^{-6}	4.3×10^{-5}	4.4×10^{-2}
fabp2	0.39	0.85	0.16	0.12	3.7×10^{-2}	3.5×10^{-3}	0.43	0.53	4.2×10^{-2}	0.52	0.09	0.28	0.61
apoa1a	0.67	0.19	0.13	0.13	6.7×10^{-3}	8.5×10^{-4}	0.11	0.57	0.69	0.55	0.06	1.4×10^{-3}	0.11
p53	0.21	0.22	0.94	3.6×10^{-3}	4.7×10^{-2}	1.7×10^{-5}	0.35	0.08	1.3×10^{-5}	4.2×10^{-2}	3.8×10^{-4}	2.0×10^{-5}	3.1×10^{-5}
p21	0.80	0.52	0.44	0.84	0.10	0.19	0.38	0.92	0.24	0.66	0.22	0.21	0.39
p16	0.34	n.c.	n.c.	n.c.	n.c.	n.c.	n.c.	0.36	0.66	n.c.	n.c.	n.c.	n.c.
hk1	0.87	0.12	0.80	0.60	0.83	0.46	0.64	0.41	0.21	0.95	0.21	0.18	0.19
pcca	0.06	0.84	0.99	0.64	0.06	0.94	0.22	0.09	0.44	0.92	0.11	0.77	0.71
cs	0.34	0.31	0.02	0.97	0.40	0.28	0.41	0.52	0.33	0.30	5.3×10^{-3}	0.39	0.43
acadm	0.67	0.25	0.34	0.51	0.08	0.20	0.61	0.98	0.02	0.60	5.2×10^{-3}	0.60	0.12
atg9a	0.31	0.13	0.73	0.29	0.99	1.4×10^{-4}	0.15	0.81	0.82	0.45	0.96	1.6×10^{-3}	0.06
atg14	0.18	0.27	0.35	0.25	0.61	6.7×10^{-3}	0.27	0.30	0.94	0.27	0.96	0.14	0.28
optn	0.75	0.80	0.47	0.24	0.06	4.1×10^{-3}	0.34	0.81	0.08	0.50	0.01	5.2×10^{-2}	0.73
sqstm1	0.78	0.74	1.0	0.17	0.34	6.1×10^{-4}	0.25	0.86	0.53	0.72	0.42	8.6×10^{-4}	0.24
bnip3lb	0.01	0.07	1.8×10^{-3}	0.46	0.09	5.4×10^{-4}	0.01	0.22	0.66	0.64	7.6×10^{-3}	3.4×10^{-3}	0.17
fis1	0.88	0.34	0.37	0.90	0.19	3.9×10^{-3}	0.40	0.93	0.19	0.65	0.50	1.6×10^{-4}	0.80
gclm	0.56	1.0	0.47	0.11	0.04	4.9×10^{-3}	0.67	0.15	0.10	0.37	0.60	0.96	0.40
txn	0.91	0.03	4.5×10^{-2}	0.42	1.5×10^{-4}	0.01	0.43	0.14	0.38	0.14	0.44	0.03	0.36

n.c. = not calculable

Ujibe et al. Table S6

Table S6. Mean ± SD of *ptf1a*:EGFP⁺ area of *wrn*$^{+/+}$, *wrn*$^{-/-}$_mild, and *wrn*$^{-/-}$_severe larvae at each stage or feeding condition.

ptf1a:EGFP^{+} area (μm^2)	*wrn*$^{+/+}$	*wrn*$^{-/-}$_mild	*wrn*$^{-/-}$_severe
5 dpf	25,538 ± 5,427	26,103 ± 4,172	
7 dpf	32,299 ± 7,200	19,642 ± 4,141	11,177 ± 3,097
9 dpf (Normal feed condition)	31,303 ± 5,499	21,556 ± 6,392	5,388 ± 2,491
12 dpf	38,907 ± 9,393	24,603 ± 6,428	3,192 ± 1,509
9dpf (Overfeed condition)	29,383 ± 6,241	26,279 ± 5,633	10,072 ± 3,679

Ujibe et al. Table S7

Table S7. Mean ± SD of villi length and intestine area of *wrn*$^{+/+}$, *wrn*$^{-/-}$_mild, and *wrn*$^{-/-}$_severe larvae at 9 dpf.

	wrn$^{+/+}$	*wrn*$^{-/-}$_mild	*wrn*$^{-/-}$_severe
Villi length (μm)	20.6 ± 3.10	18.1 ± 4.89	14.7 ± 3.16
Intestine size (μm^2)	9,904 ± 2,851	6,905 ± 2,362	3,740 ± 2,325

Ujibe et al. Table S8

Table S8. Mean ± SD of γH2AX⁺, EdU⁺, and TUNEL⁺ cells of *wrn*$^{+/+}$, *wrn*$^{-/-}$_mild, and *wrn*$^{-/-}$_severe larvae at each stage.

	Tissue	(dpf)	*wrn*$^{+/+}$	*wrn*$^{-/-}$_mild	*wrn*$^{-/-}$_severe
γH2AX^{+} cells	Pancreas	5	0	29.4 ± 10.5	
		7	0.17 ± 0.41	21.0 ± 14.7	6.50 ± 4.55
	Intestine	5	0	51.8 ± 23.7	
		7	7.83 ± 3.87	72.8 ± 20.2	42.8 ± 22.1
EdU^{+} cells	Pancreas	5	6.75 ± 2.75	5.33 ± 3.88	
		7	8.20 ± 4.87	7.67 ± 3.93	0.43 ± 0.78
	Intestine	5	47.3 ± 15.4	53.5 ± 18.0	
		7	59.0 ± 26.1	24.2 ± 17.0	3.43 ± 5.53
TUNEL^{+} cells	Pancreas	5	0	0	
		7	0.17 ± 0.41	0.83 ± 1.33	4.00 ± 2.75
	Intestine	5	0.20 ± 0.44	0.80 ± 1.30	
		7	1.00 ± 2.00	1.00 ± 1.55	2.33 ± 1.75

Ujibe et al. Table S9

Table S9. Mean ± SD of telomere length of young and old zebrafish, and *wrn*$^{+/+}$, *wrn*$^{-/-}$_mild, and *wrn*$^{-/-}$_severe larvae at 7 dpf.

	young	old	*wrn*$^{+/+}$	*wrn*$^{-/-}$_mild	*wrn*$^{-/-}$_severe
Telomere length	1.00 ± 0.05	0.90 ± 0.02	1.05 ± 0.32	0.89 ± 0.03	0.91 ± 0.02

Ujibe et al. Table S10

Table S10. Mean ± SD of glucose and glycogen of *wrn*$^{+/+}$, *wrn*$^{-/-}$_mild, and *wrn*$^{-/-}$_severe larvae at 9 dpf.

	wrn$^{+/+}$	*wrn*$^{-/-}$_mild	*wrn*$^{-/-}$_severe
Glucose (ng)	51.4 ± 29.6	43.2 ± 16.8	25.2 ± 11.8
Glycogen (ng)	54.9 ± 30.3	37.4 ± 19.5	14.3 ± 5.44

Ujibe et al. Table S11

Table S11. Mean ± SD of lipid intensity of *wrn*$^{+/+}$, *wrn*$^{-/-}$_mild, and *wrn*$^{-/-}$_severe larvae at 9 dpf.

	wrn$^{+/+}$	*wrn*$^{-/-}$_mild	*wrn*$^{-/-}$_severe
Lipid intensity	1.00 ± 0.03	0.90 ± 0.07	0.87 ± 0.05
